# Response to neoadjuvant chemotherapy in early breast cancers is associated with epithelial–mesenchymal transition and tumor‐infiltrating lymphocytes

**DOI:** 10.1002/1878-0261.13813

**Published:** 2025-02-06

**Authors:** Françoise Derouane, Jérôme Ambroise, Cédric van Marcke, Mieke Van Bockstal, Martine Berlière, Christine Galant, Hélène Dano, Médina Lougué, Elena Benidovskaya, Guy Jerusalem, Vincent Bours, Claire Josse, Jérôme Thiry, Aurélie Daumerie, Caroline Bouzin, Cyril Corbet, François P. Duhoux

**Affiliations:** ^1^ Department of Medical Oncology University Hospital Leuven Belgium; ^2^ Pole of Medical Imaging, Radiotherapy and Oncology (MIRO) Institut de Recherche Expérimentale et Clinique (IREC), UCLouvain Brussels Belgium; ^3^ Center for Applied Molecular Technologies (CTMA) Institut de Recherche Expérimentale et Clinique (IREC), UCLouvain Brussels Belgium; ^4^ Department of Medical Oncology Institut Roi Albert II, Cliniques Universitaires Saint‐Luc Brussels Belgium; ^5^ Department of Pathology Cliniques Universitaires Saint‐Luc Brussels Belgium; ^6^ Pole of Morphology (MORF) Institut de Recherche Expérimentale et Clinique (IREC), UCLouvain Brussels Belgium; ^7^ Pole of Gynecology (GYNE) Institut de Recherche Expérimentale et Clinique (IREC), UCLouvain Brussels Belgium; ^8^ Department of Gynecology Institut Roi Albert II, Cliniques Universitaires Saint‐Luc Brussels Belgium; ^9^ Department of Medical Oncology University Hospital (CHU) Liège Belgium; ^10^ Faculty of Medicine University of Liège Belgium; ^11^ Laboratory of Human Genetics, GIGA Institute University of Liège Belgium; ^12^ Center of Genetics University Hospital (CHU), University of Liège Belgium; ^13^ 2IP Imaging Platform Institut de Recherche Expérimentale et Clinique (IREC), UCLouvain Brussels Belgium; ^14^ Pole of Pharmacology and Therapeutics (FATH) Institut de Recherche Expérimentale et Clinique (IREC), UCLouvain Brussels Belgium

**Keywords:** early breast cancer, EMT, lymphocytes, neoadjuvant chemotherapy, TILs

## Abstract

Epithelial–mesenchymal transition (EMT) and tumor‐infiltrating lymphocytes (TILs) play a central role in early‐stage breast cancer (BC) and are associated with chemoresistance, stemness, and invasion. The objective of this study was two fold: (a) by investigating the predictive value of EMT and TILs, we aimed to estimate the chance of achieving a response after neoadjuvant chemotherapy (NAC) and (b) to evaluate the potential changes of EMT and TILs in BC upon NAC. Using bulk RNA sequencing and immunofluorescence (IF) for EMT (E‐cadherin and vimentin) and lymphocyte markers (CD3, CD8, FOXP3), we analyzed pre‐ and post‐NAC tumor samples from 100 early‐BC patients treated with NAC. For each BC molecular subtype, we compared the expression of EMT and TILs, at the RNA and protein level, between responding and non‐responding tumors. Paired analysis of pre‐ and post‐NAC samples was performed for patients with residual disease after NAC. RNA sequencing of pre‐ and post‐NAC samples identified significant differences in EMT‐related and inflammation‐related gene expression between non‐responding (RCB‐II/III) and responding (RCB‐0/I) tumors. Increased EMT‐related marker expression was observed after NAC in cases with residual disease, in particular in the luminal subtype. Characterization of TILs in pre‐NAC samples showed substantially more CD3 + CD8‐FOXP3‐lymphocytes in responding HER2+ tumors compared with non‐responding. Paired analyses of pre‐ and post‐NAC samples demonstrated higher levels of CD3 + CD8 + FOXP3‐lymphocytes in residual luminal and triple‐negative BC and higher levels of CD3 + CD8‐FOXP3‐lymphocytes in residual triple‐negative BC compared with other subtypes of lymphocytes. We found that there is an unmet clinical need for reliable biomarkers to predict response to NAC in BC. Our results suggest that an upregulation of the EMT gene signature in diagnostic biopsies is associated with poor response to NAC in early BC, across all subtypes. Additionally, changes in EMT and in the TIL population occur in residual tumors after NAC. These findings could help to personalize future NAC and adjuvant treatment regimens.

## Graphical abstract

Epithelial–mesenchymal transition (EMT) and tumor‐infiltrating lymphocytes (TILs) are associated with early breast cancer response to neoadjuvant chemotherapy (NAC). This study evaluated EMT and TIL shifts, with immunofluorescence and RNA sequencing, at diagnosis and in residual tumors as potential biomarkers associated with treatment response. Our findings highlight the need for reliable biomarkers to guide personalized NAC regimens across BC subtypes.

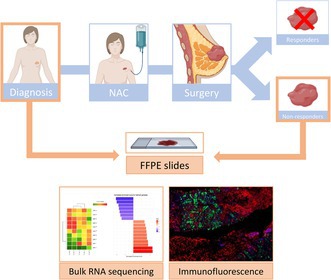

**Abbreviations**
ALND: axillary lymph node dissection
BC: breast cancer
BCS: breast conserving surgery
BSA: bovine serum albumine
CI: confidence interval
DAB: diaminobenzidine
DEG: differentially expressed genes
EC: epirubicin and cyclophosphamide
EFS: event‐free survival
EMT: epithelial–mesenchymal transition
ES: enrichment score
FEC: 5‐fluorouracile, epirubicin, cyclophosphamide
FFPE: formalin‐fixed paraffin‐embedded
GSEA: gene set enrichment analysis
HE: hematoxylin – eosin
HRP: horseradish peroxidase
IDC: invasive ductal carcinoma
ILC: invasive lobular carcinoma
NAC: neoadjuvant chemotherapy
OR: odds ratio
OS: overall survival
pCR: pathological complete response
RCB: residual cancer burden
RT: room temperature
SLNB: sentinel lymph node biopsy
T: taxanes
TBS: tris buffered saline
TC: taxanes, carboplatin
TILs: tumor‐infiltrating lymphocytes
TNBC: triple negative breast cancer
TSA: tyramide signal amplification

## 1 Introduction

The majority of breast cancers (BCs) are diagnosed at an early stage and can be cured with multimodal treatments [1]. In high‐risk early BC, neoadjuvant chemotherapy (NAC) followed by surgery is the standard of care. Pathological complete response (pCR) to NAC has been validated as a prognostic marker with improved event‐free survival (EFS) and overall survival (OS) and is currently evaluated by the residual cancer burden (RCB) score [2, 3, 4, 5]. Further optimization of the treatment in the neoadjuvant setting requires a reliable marker enabling to predict the response to NAC in patients with early BC, to allow a better selection of patients requiring treatment escalation strategies, but also to reduce the burden of toxicity in patients who already derive high benefit from conventional treatments [6]. As an example, in locally advanced triple‐negative breast cancer (TNBC), the addition of pembrolizumab to the NAC backbone of anthracyclines, taxanes, and carboplatin is now standard of care, with 65% of patients achieving a pCR. However, anthracycline/taxane‐based NAC without immunotherapy already leads to a 50% pCR rate, and recurrence rates do not differ according to the means of achieving pCR [7]. Immunotherapy could thus be avoided in 50% of patients, highlighting the unmet medical need of reliable biomarkers to predict response to NAC in BC patients.

A better understanding of the mechanisms associated with resistance to NAC would allow to the development of new strategies to improve the clinical management of these patients [6].

Epithelial–mesenchymal transition (EMT) has been described as a major phenotypic trait in cancer cells, including BC. EMT is a dynamic and reversible phenomenon in which cells gradually lose their epithelial features and develop mesenchymal traits, acquiring properties to invade and colonize secondary sites along a continuum of phenotypes [8]. In BC, EMT has been reported to support chemoresistance, immune evasion, and promote invasiveness [9]. The prognostic role of immune cells in the microenvironment, and more specifically tumor‐infiltrating lymphocytes (TILs), has also been extensively studied in early BC [10]. Several trials have shown a correlation between high levels of TILs and pCR in all BC subtypes, but despite being promising, TILs assessment does not seem mature enough to be used in clinical practice as a predictive biomarker [6, 11]. In this trial, we studied the association between EMT and TILs infiltration and response to NAC using bulk RNA sequencing analysis and immunofluorescence (IF). Using these two techniques, we have evaluated the predictive value of these markers as well as their potential modulation after exposure to NAC across all BC subtypes. In this original work, we were able to highlight the relevance of EMT and TILs evaluation in early BC.

## 2 Materials and methods

### 2.1 Study design and data collection

The study is a monocentric and observational trial in which patients with early BC, eligible for NAC, were enrolled regardless of the molecular subtype. Patients were recruited, retrospectively and prospectively, at Cliniques universitaires Saint‐Luc (CUSL; Brussels, Belgium) and were diagnosed with early BC from 2012 to 2022. For the retrospective cohort, recruited from 2012 to 2017, no informed consent was needed, while for the prospective cohort, recruited from 2018 to 2022, subjects gave informed consent. All tumor specimens were obtained from the department of pathology at CUSL. The collected tumor samples comprised formalin‐fixed, paraffin‐embedded (FFPE) tissue slides from diagnostic biopsies (pre‐NAC) and surgical resection specimens in non‐responding tumors (post‐NAC). Pathological and clinical information from this cohort was collected and managed using the REDCap (Research Electronic DataCapture) secured database hosted at CUSL, with data anonymization [12, 13]. The study methodologies conformed to the standards set by the Declaration of Helsinki. The experiments were undertaken with the understanding and written consent of each subject in the prospective study. The study methodologies were approved by the local ethics committee, “Comité d'éthique hospitalo‐facultaire,” CEHF – Ethics committee number: 2017/25JUL/376. Trial registration: NCT03314870.

### 2.2 Type of analyses performed on the pre‐ and post‐NAC BC samples

**Fig. 1:**
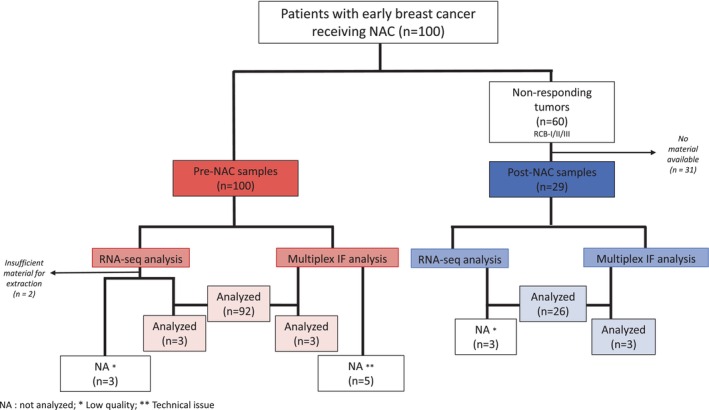
CONSORT diagram detailing the analyses performed on the pre‐ and post‐NAC BC samples. In pre‐NAC samples, RNA‐seq analysis was performed in 95 samples due to the exclusion of 5 samples for insufficient material before extraction (*n* = 2) or low quality extracted RNA (*n* = 3). For multiplex IF analyses from pre‐NAC samples, 5 samples were excluded due to technical issues. In post‐NAC samples, RNA‐seq analysis was possible in 26 samples due to the exclusion of 3 samples for low quality extracted RNA. Multiplex IF was done and analyzed in all the post‐NAC samples (*n* = 29). BC, breast cancer; IF, immunofluorescence; NAC, neoadjuvant chemotherapy.

A CONSORT diagram details the analyses performed on the pre‐ and post‐NAC samples (Fig. 1). Among 100 recruited patients, 98 pre‐NAC tumor samples contained sufficient tumor tissue to allow RNA extraction and sequencing. Specimens from 3 patients were excluded from the analysis due to poor sequencing quality and/or DNA contamination. In total, gene expression data from 95 pre‐NAC samples were analyzed. For the multiplex analysis by IF, 95 pre‐NAC samples were available, after the exclusion of 5 samples due to technical issues. Out of the 60 non‐responding tumors, 29 post‐NAC samples were also obtained from BC patients with residual disease (RCB I‐II and III) and used for RNA sequencing and IF analysis. In total, we performed concomitant RNA‐sequencing and multiplex IF analyses on 92 diagnostic biopsies and 26 post‐NAC surgical resections with residual disease.

### 2.3 RNA extraction, library preparation, and RNA sequencing

The extraction protocol, library preparation, and RNA sequencing have already been explained by Derouane et al. [14] Fifteen to twenty 5 μm‐thick FFPE sections from diagnostic biopsies were cut. A pathologist delineated the tumor zone on a hematoxylin/eosin‐stained slide, which was subsequently identified on the other unstained slides for macro‐dissection. After a brief centrifugation, deparaffinization and extraction steps were carried out using the AllPrep DNA/RNA FFPE kit (#80234; Qiagen, Antwerp, Belgium), according to the manufacturer's recommendations, and DNA was removed from the samples with the TURBO DNA‐free™ kit (#AM1907; Thermo Fisher Scientific, Waltham, MA, USA), by following the manufacturer's instructions. Total RNA samples were then quantified by using Quant‐it™ RiboGreen RNA Assay kit (#R11490; Thermo Fisher Scientific), before ribosomal RNA depletion with the NEBNext rRNA Depletion kit (Human/Mouse/Rat) with sample purification beads (#E6350; New England Biolabs, Ipswich, MA, USA). Libraries were prepared with the NEBNext Ultra II Directional RNA Library Prep with Beads kit (#E7765; New England Biolabs). Adaptors and primers used for the library preparation were from NEBNext Multiplex Oligos for Illumina (Dual Index Set 1), 96 rxns (#E7760; New England Biolabs). The quality of the libraries was evaluated using a High Sensitivity DNA kit (chips and reagents) (#5067 and #4626; Agilent, Machelen, Belgium) with a 2100 Bioanalyzer system (Agilent). Sequence reads were generated on the Illumina NovaSeq 6000 sequencer on an S4 cartridge (300 bp). RNA sequencing was performed in 3 distinct batches.

### 2.4 Bioinformatics analyses

Raw NGS sequences were filtered to remove low‐quality reads. The quality of analyzed data was checked using FastQC and QualiMap, while trimming was carried out by Trimmomatic. Filtered data were then mapped by CLC Genomics v22 software (Qiagen) to the *Homo sapiens* genome hg38 and the RNA database v91. On average, 2 × 10^6^ reads could be mapped to the human genome. Samples with <500 000 mapping reads were excluded. The read count expression matrix was analyzed using the edger v3.40.2 Bioconductor package in order to detect differentially expressed genes (DEGs) [15]. Briefly, a filtering strategy was first applied in order to keep genes having sufficiently large counts to be retained in statistical analyses. Scaling factors were then computed with the trimmed mean of M‐values (TMM) method. A quasi‐likelihood negative binomial generalized log‐linear model was built on the resulting data. In addition to the variable of interest (e.g., RCB), a batch effect, a DNA contamination effect (estimated by the percentage of intergenic mapping), as well as the age of the patients, were explicitly incorporated in the models in order to correct for the potential confounding effects of these factors, as recommended by Gallego Romero et al. [16]. This modeling strategy was first applied to detect DEGs between non‐responding and responding tumors (RCB II‐III vs. 0‐I) for each molecular subtype (i.e., stratified analysis for Luminal, HER2+, and TNBC). This stratified analysis was also applied to detect genes with expression levels significantly correlated with EMT markers (E‐cadherin and vimentin) measured with multiplex IF (see below). Finally, based on the resulting gene expression modulations, gene set enrichment analysis (GSEA) was performed using the fgsea v1.24.0 [17] bioconductor packages on hallmark gene sets of the MSigDB collection [18]. Two publicly available transcriptomic datasets, GSE22226 and GSE25066, were also analyzed to confirm our findings in the three breast cancer subtypes. Gene expression profiles from the GSE22226 dataset were obtained using Agilent‐012391 Whole Human Genome Oligo Microarray G4112A, and profiles from the GSE25066 dataset were obtained using the Affymetric Human Genome U133A Array. For both datasets, metadata was imported using the geoquery v.2.66.0 Bioconductor package. For the GSE22226 dataset, we selected 23 patients with luminal B breast cancer, 20 patients with HER2+ breast cancer, and 39 patients with triple negative, while in the GSE25066, 248 patients with luminal breast cancer and 146 patients with triple negative breast cancer were analyzed. Patient's selection was based on the availability of pathological response information (RCB) but also on the NAC regimen (in both datasets: anthracyclines and taxanes). The limma Bioconductor package was used to estimate fold change and *P*‐values associated with pathological response (RCB) while adjusting for patient age. GSEA was conducted in both datasets using the fgsea v1.24.0 Bioconductor package on hallmark gene sets from the MSigDB collection.

### 2.5 Multiplex IF – immunohistochemistry

Expression of vimentin, E‐cadherin, CD3, CD8, and FOXP3 was assessed on 4‐μm FFPE section biopsies by following previously published protocols [19, 20] (Fig. S1). Briefly, endogenous peroxidases were inhibited for 20 min with 3% hydrogen peroxide in methanol. Sections were then subjected to antigen retrieval in 1 mm citrate buffer pH 5.7 and to blocking of aspecific antigen binding sites (Tris buffered saline (TBS) + Bovine Serum Albumine (BSA) 5% + 0.1% Tween 20). The first primary antibody was incubated 1 h at room temperature (RT) in TBS containing 1% BSA and 0.1% Tween 20 and detected by corresponding horseradish peroxidase (HRP)‐conjugated secondary antibodies for 1 h at RT. HRP was then visualized by tyramide signal amplification (TSA) using AlexaFluor‐conjugated tyramides (Thermo Fisher Scientific or homemade). After a new citrate incubation step, the same protocol was applied with other primary antibodies and different AlexaFluor‐ or Atto‐conjugated tyramides. In this study, 5 sequential incubations were performed as indicated in Table S1. After a washing step in PBS, nuclei were finally stained with Hoechst 33342 (Thermo Fisher Scientific) diluted in TBS containing 10% BSA and 0.1% Tween 20, washed in TBS containing 0.1% Tween 20, and mounted with Dako fluorescence mounting medium (Agilent). Slides were stored at −20°C until multispectral image acquisition with a Pannoramic 250 Flash III slide scanner (3DHistech) at ×20 magnification. After the fluorescence image acquisition, coverslips were removed by immersion of the slides into water overnight at RT in order to stain the tumor area in brightfield on the same section. After an additional step of citrate buffer incubation, anti‐pan‐cytokeratin antibody was incubated for 1 h at RT, followed by HRP‐conjugated secondary antibody. Peroxidase activity was revealed upon incubation for 5 min with diaminobenzidine (DAB) substrate (Agilent #K3468). Slides were finally counterstained for 3 min with hematoxylin (Agilent #S3301), mounted with a Dako coverslipper, and digitalized using the same slide scanner.

### 2.6 Evaluation of TILs

The extent of the stromal tumor‐infiltrating lymphocytes (sTILs) in the pre‐NAC biopsy and the post‐NAC resection specimens was assessed according to the standardized method as described by the International Immuno‐oncology Biomarkers Working Group [11]. The number of sTILs was noted as the percentage of mononuclear inflammatory cells related to the total peri‐ and intra‐tumor stromal surface area, which served as a denominator. sTILs evaluation was performed by two pathologists (MVB and ML) using a multi‐head microscope to obtain a consensus diagnosis.

### 2.7 Computer‐assisted quantitative evaluation of immunostaining in whole tissue sections

Stains were quantified on multiplex‐stained paraffin sections of entire tissue sections with software applications (“APP”s) using the image analysis tool Author version 2017.2 (Visiopharm, Hørsholm, Denmark). Fluorescent and brightfield scans were first aligned for each slide using the TissueAlign add‐on of the software. On the brightfield scan, tumor regions were manually circled by a pathologist and then automatically adjusted to the tissue borders by a first APP. Tumor clusters (pan‐CK+) and stromal tissue (pan‐CK−) were then automatically delineated by a second APP. Manual correction was applied when required to discard folds and blurred areas. Tumor and stroma regions detected on the brightfield scan were transposed to the aligned fluorescent scans. Cells were then detected at high resolution (×20) with a nuclear‐based cell segmentation relying on Hoechst staining. Following segmentation, post‐processing steps were applied to classify cells according to the fluorescence intensity of each marker. EMT markers were quantified only in the tumor clusters, while immune markers were detected in the tumoral and stromal compartment region. Results were expressed as percentage of stained cells. To assess E‐cadherin and vimentin intensity within tumor clusters, a staining index was calculated for each marker as the percentage of stained area over a fixed threshold multiplied by the mean intensity within this stained area. For spatial analysis, the number of each cell type at a maximal distance of 25 and 50 μm from tumor clusters was quantified. Results were expressed as percentage of stained cells.

### 2.8 Statistical analyses

T‐test and non‐parametric Wilcoxon test (in case of small sample size (<30 samples) and non‐normal distribution) were used. Chi‐square test and multiple logistic regression models were used to analyze the factors associated with response (RCB II‐III vs. 0‐I). The comparison of EMT protein expression levels between pre‐ and post‐NAC samples was performed using mixed effect models with a random patient effect and a ‘time’ fixed effect. In the multivariate model analysis of clinical data, variable selection was performed using a backward elimination and following the recommendations of Vittinghoff et al. [21]. The correlation between continuous variables was assessed using Pearson's correlation coefficient. All statistical analyses were performed using the R software (4.3.1).

### 2.9 Neoadjuvant regimen

Patients received the NAC regimen depending on the BC subtype, their comorbidities, and the recommendations at the time of their diagnosis. They could receive: (a) 4 cycles of EC (epirubicin 90 mg/m^2^ and cyclophosphamide 600 mg/m^2^ intravenously (iv)) followed by 12 cycles of weekly paclitaxel (80 mg/m^2^ iv), (b) 4 cycles of FEC (5‐fluorouracil 500 mg/m^2^, epirubicin 100 mg/m^2^, cyclophosphamide 500 mg/m^2^ iv) followed by 4 cycles of docetaxel (75 mg/m^2^ iv), (c) a combination of docetaxel (75 mg/m^2^ iv) and cyclophosphamide (600 mg/m^2^ iv) for 4 to 6 cycles, or (d) a sequential treatment of 4 cycles of EC (epirubicin 90 mg/m^2^ and cyclophosphamide 600 mg/m^2^ iv) followed by 12 cycles of paclitaxel (80 mg/m^2^ iv) associated with carboplatin (AUC 5, iv). In case of HER2‐positive status, and depending on the recommendations at the time of diagnosis, trastuzumab (6 mg/kg iv) with or without pertuzumab (420 mg, fixed dose, iv) was administered. It should be noted that none of the patients included in our analyses received immunotherapy.

### 2.10 Evaluation of response to NAC

Most patients received sequential treatment of anthracyclines followed by taxanes. Response to NAC was evaluated by histological examination of the surgical resection specimen, sliced at 5 mm intervals. Pathological complete response (pCR) was defined as the absence of an invasive tumor in the breast and in the axilla, with the calculation of the residual cancer burden score (RCB), as previously described [5, 22]. RCB classes of 0 (no residual disease) or I (minimal residual disease) were considered responding tumors, while RCB classes of II (moderate residual disease) or III (extensive residual disease) were considered non‐responding tumors. The study cohort comprised 40 RCB‐0 patients (luminal 5/40, HER2+ 19/40, TNBC 16/40), 20 RCB‐I patients (luminal 6/20, HER2+ 9/20, TNBC 5/20), 25 RCB‐II patients (luminal 15/25, HER2+ 6/25, TNBC 4/25) and 15 RCB‐III patients (luminal 8/15, HER2+ 3/15, TNBC 4/15).

## 3 Results

### 3.1 Association between clinicopathological parameters of the patients and response to NAC

The main clinical characteristics of the cohort are described in Table 1. Of the 100 women diagnosed with early BC and treated with NAC, 60 were considered responders (RCB‐0 and I) and 40 as non‐responders (RCB‐II and III). In both groups, invasive breast cancer of no special type (IBC‐NST) was the main histological subtype. Tumor size at diagnosis was not significantly different between the 2 groups (*P*‐value 0.15). In all patients from the responder group, Ki67 was higher than 15% at diagnosis. As compared to non‐responding tumors, responding tumors were statistically significantly enriched in grade 3 tumors, unifocal lesions, and TNBC subtypes (*P*‐values 0.009, 0.02, and 0.0002 respectively). The majority of the patients received sequential treatment of anthracyclines followed by taxanes. A multivariate logistic regression model further identified unifocal lesions and molecular subtypes as the main variables impacting the response to NAC in BC patients (Table 2). Unifocal lesions were associated with a decreased risk (OR = 0.36) of non‐response. Similarly, as compared to luminal cases, HER2+ and TNBC subtypes were associated with a decreased risk (OR = 0.15 and 0.23, respectively) of non‐response.

**Table 1:** Clinicopathological parameters for patients with early BC enrolled in our study. Responding patients are RCB 0‐I, and non‐responding patients are RCB II‐III. ALND, axillary lymph node dissection; BCS, breast‐conserving surgery; EC‐T, epirubicin/cyclophosphamide‐taxane; EC‐T‐C, epirubicin/cyclophosphamide‐taxane‐carboplatin; FEC, 5‐FU, epirubicin, cyclophosphamide; IBC‐NST, invasive breast cancer of no special type; ILC, invasive lobular carcinoma; SLNB, sentinel lymph node biopsy; TC, taxane, carboplatin.

Characteristics	All patients (*n* = 100)	Responders (RCB‐0/I, *n* = 60)	Non‐responders (RCB‐II/III, *n* = 40)	*P*‐value^a^
Age	53.6 ± 12.6	54.0 ± 13.3	53.0 ± 11.7	0.70
Menopausal status				0.96
Non‐menopausal	41 (41%)	24 (40%)	17 (42%)	
Menopausal	59 (59%)	36 (60%)	23 (58%)	
Histological type				0.15
IBC‐NST	92 (92%)	58 (97%)	34 (85%)	
ILC	7 (7%)	2 (3%)	5 (13%)	
Mixed carcinoma	1 (1%)	0	1 (2%)	
Tumor size (largest diameter – in mm)	36.0 ± 24.5	33 ± 21.0	40.6 ± 28.8	0.15
Unifocal				**0.02** *
Yes	55 (55%)	33 (55%)	12 (30%)	
No	45 (45%)	27 (45%)	28 (70%)	
Grade				**0.009** **
I	2 (2%)	0	2 (5%)	
II	25 (26%)	10 (17%)	15 (37%)	
III	73 (73%)	50 (83%)	23 (58%)	
Ki67				**0.007** **
<15%	6 (6%)	0	6 (15%)	
≥15%	94 (94%)	60 (100%)	34 (85%)	
Nodal invasion at diagnosis				0.21
Yes	64 (64%)	35 (58%)	29 (72%)	
No	36 (36%)	25 (42%)	11 (28%)	
Molecular subtype				**0.0002** ***
Luminal	34 (34%)	11 (18%)	23 (58%)	
HER2+	37 (37%)	28 (47%)	9 (22%)	
TNBC	29 (29%)	21 (35%)	8 (20%)	
Type of NAC				**0.04** *
FEC	3 (3%)	0	3 (8%)	
TC	2 (2%)	1 (2%)	1 (2%)	
EC‐T	93 (93%)	59 (98%)	34 (85%)	
EC‐T‐C	2 (2%)	0	2 (5%)	
Anti‐HER2 therapy				**0.02** *
Yes	37 (37%)	28 (47%)	9 (22%)	
No	63 (63%)	32 (53%)	31 (78%)	
Type of breast surgery				**0.03** *
BCS	49 (49%)	35 (58%)	14 (35%)	
Mastectomy	51 (51%)	25 (42%)	26 (65%)	
Type of axillary surgery				**0.009** **
SLNB	25 (25%)	21 (35%)	4 (10%)	
ALND	75 (75%)	39 (65%)	36 (90%)	
Adjuvant radiotherapy				1
Yes	85 (85%)	51 (85%)	34 (85%)	
No	15 (15%)	9 (15%)	6 (15%)	
Adjuvant endocrine therapy				**0.0004** ***
Yes	55 (55%)	24 (40%)	31 (77%)	
No	45 (45%)	36 (60%)	9 (23%)	

a Continuity‐corrected chi‐square test and t‐test for categorical and continuous variables, respectively.

Asterisks denote the following levels of statistical significance: *P < 0.05, **P < 0.01, and ***P < 0.001.

**Table 2:** Multivariate analysis of clinicopathological variables in BC patients. Multivariate analysis by logistic regression of clinicopathological parameters depending on response status (RCB 0‐I vs. RCB II‐III) in BC patients.

Variables	Odds ratio	Confidence interval (97.5%)	*P*‐value
Menopausal status	1.02	0.99–1.04	0.13
Tumor size (mm)	1.36	0.53–3.65	0.53
Unifocal	0.36	0.13–0.94	**0.04** *
Nodal invasion at diagnosis	1.42	0.51–4.02	0.5
Molecular subtype			
Luminal	1	–	–
HER2+	0.15	0.05–0.45	**<0.001** ***
Triple‐negative	0.23	0.06–0.74	**0.02** *

Asterisks denote the following levels of statistical significance: *P < 0.05, **P < 0.01, and ***P < 0.001.

### 3.2 EMT‐related genes are upregulated in pre‐NAC samples from non‐responding BC patients across each molecular subtype

**Fig. 2:**
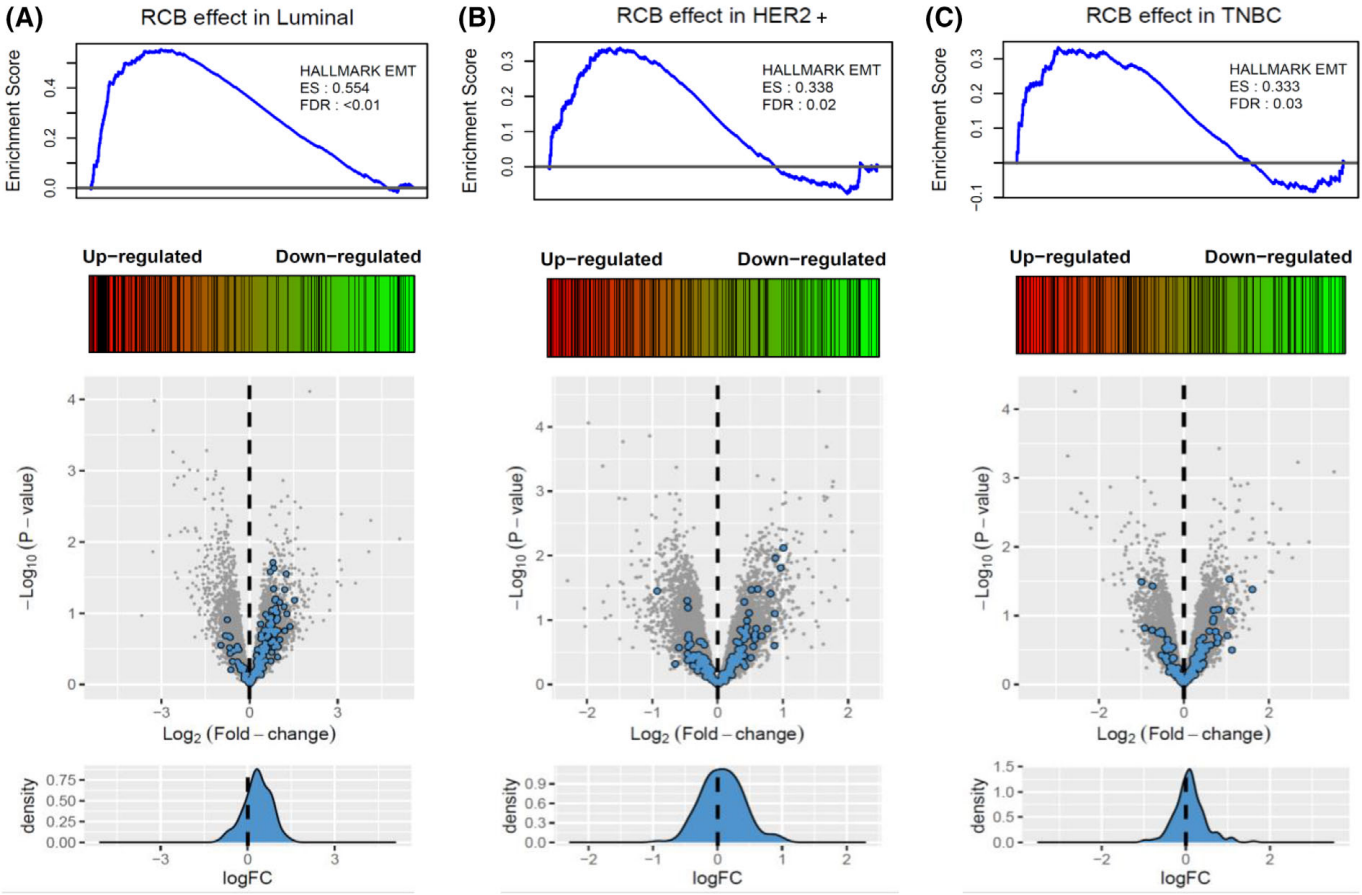
Enrichment of the EMT hallmark gene set in BC patients not responding to NAC. Enrichment plots and volcano plots for the hallmark epithelial–mesenchymal transition (EMT), comparing non‐responding (RCB II‐III) and responding (RCB 0‐I) patients with luminal (A), HER2+ (B), or triple negative (C) BC subtypes. BC, breast cancer; EMT, epithelial–mesenchymal transition; ES, enrichment score; FDR, false discovery rate; NAC, neoadjuvant chemotherapy; RCB, residual cancer burden.

Using the 95 pre‐NAC samples passing RNA sequencing quality control, no significant modulation was found by DEG analysis in each subtype between non‐responding and responding patients. However, GSEA identified the hallmark epithelial–mesenchymal transition pathway as significantly positively (Enrichment score (ES) > 0.3, FDR <0.05) enriched in each molecular subtype (Fig. 2; Tables S2– S12). These results were further validated in publicly available transcriptomic datasets, namely GSE22226 and GSE25066, with a significant positive enrichment of the hallmark epithelial–mesenchymal pathway (ES >0.3, FDR <0.05) (Fig. S2).

### 3.3 Immunodetection of EMT markers is not associated with response to NAC in diagnostic BC biopsies

**Fig. 3:**
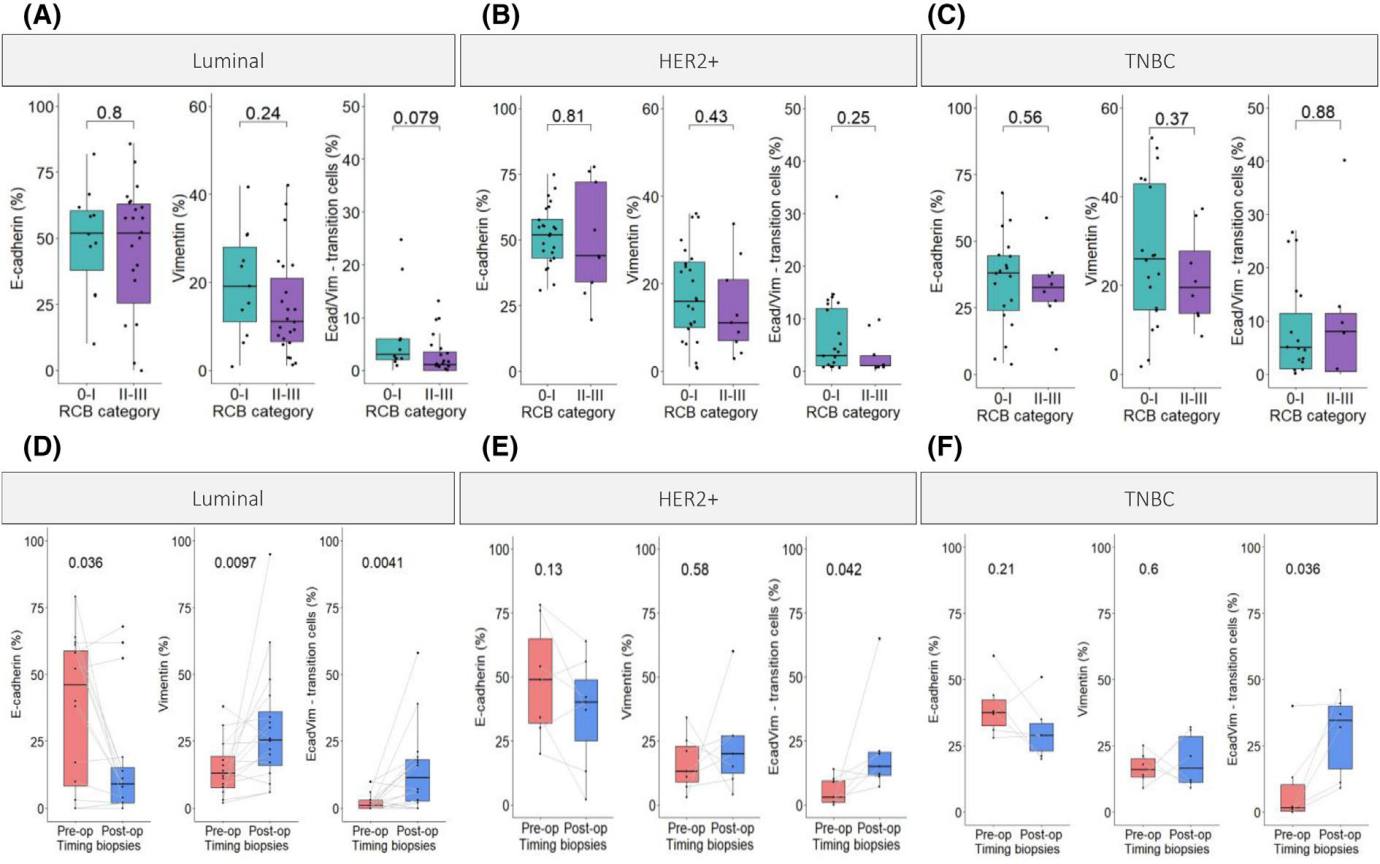
Expression levels for EMT protein markers. Comparison between responders and non‐responders (A) in luminal subtype (RCB 0‐I: *n* = 11, RCB II‐III: *n* = 23); (B) in HER2+ subtype (RCB 0‐I: *n* = 25, RCB II‐III: *n* = 9); (C) in TNBC subtype (RCB 0‐I: *n* = 19, RCB II‐III: *n* = 8). For A, B, and C: Non‐paired Wilcoxon test (±1.5 × IQR). Paired comparison of E‐cadherin, vimentin and transition cells in pre‐NAC and post‐NAC samples (*n* = 29) (D) in luminal subtype (*n* = 16); (E) in HER2+ subtype (*n* = 7); (F) in TNBC subtype (*n* = 6). For D, E, and F: Paired Wilcoxon test (±1.5 × IQR). EMT, epithelial–mesenchymal transition; NAC, neoadjuvant chemotherapy; RCB, residual cancer burden; TNBC, triple negative breast cancer.

Multiplex IF analysis was carried out on 95 pre‐NAC BC samples to assess E‐cadherin and vimentin protein expression levels and to determine the percentage of positive cells in the tumor area as well as the staining index of each protein. Cells with immunoreactivity for both vimentin and E‐cadherin markers were considered as “transition cells”. When analyzing pre‐NAC samples of each subtype separately, no difference was seen in E‐cadherin, vimentin, and transition cell percentage or staining index between responding and non‐responding tumors (Figs 3A–C, S3A,B). The results were similar when excluding the samples (*n* = 7) with invasive lobular histology (Fig. S4A–C).

### 3.4 Transition cells are observed in residual BC after NAC

Paired analyses were done between pre‐NAC samples and post‐NAC samples from surgical specimens in tumors with residual disease (RCB‐I to III, *n* = 29). We observed a decrease in the percentage of positive cells for E‐cadherin and an increase in positive cells for vimentin in the post‐NAC samples of each molecular subtype. However, these changes were only statistically significantly different in the luminal BC subtype (E‐cadherin *P*‐value 0.036, vimentin *P*‐value 0.0097) (Fig. 3D–F). The transition cells were significantly more present in post‐NAC samples of each subtype (luminal *P*‐value 0.0041, HER2+ *P*‐value 0.042, TNBC *P*‐value 0.036). When analyzing the staining index, we observed a statistically significant difference in vimentin with a higher percentage of staining index in residual tumors of luminal and TNBC subtypes (Fig. S3C,D). The results were similar when excluding the samples (*n* = 7) with invasive lobular histology (Fig. S4D–F).

### 3.5 The inflammatory response pathway is differentially regulated in diagnostic biopsies from non‐responding luminal and HER2+ BC

**Fig. 4:**
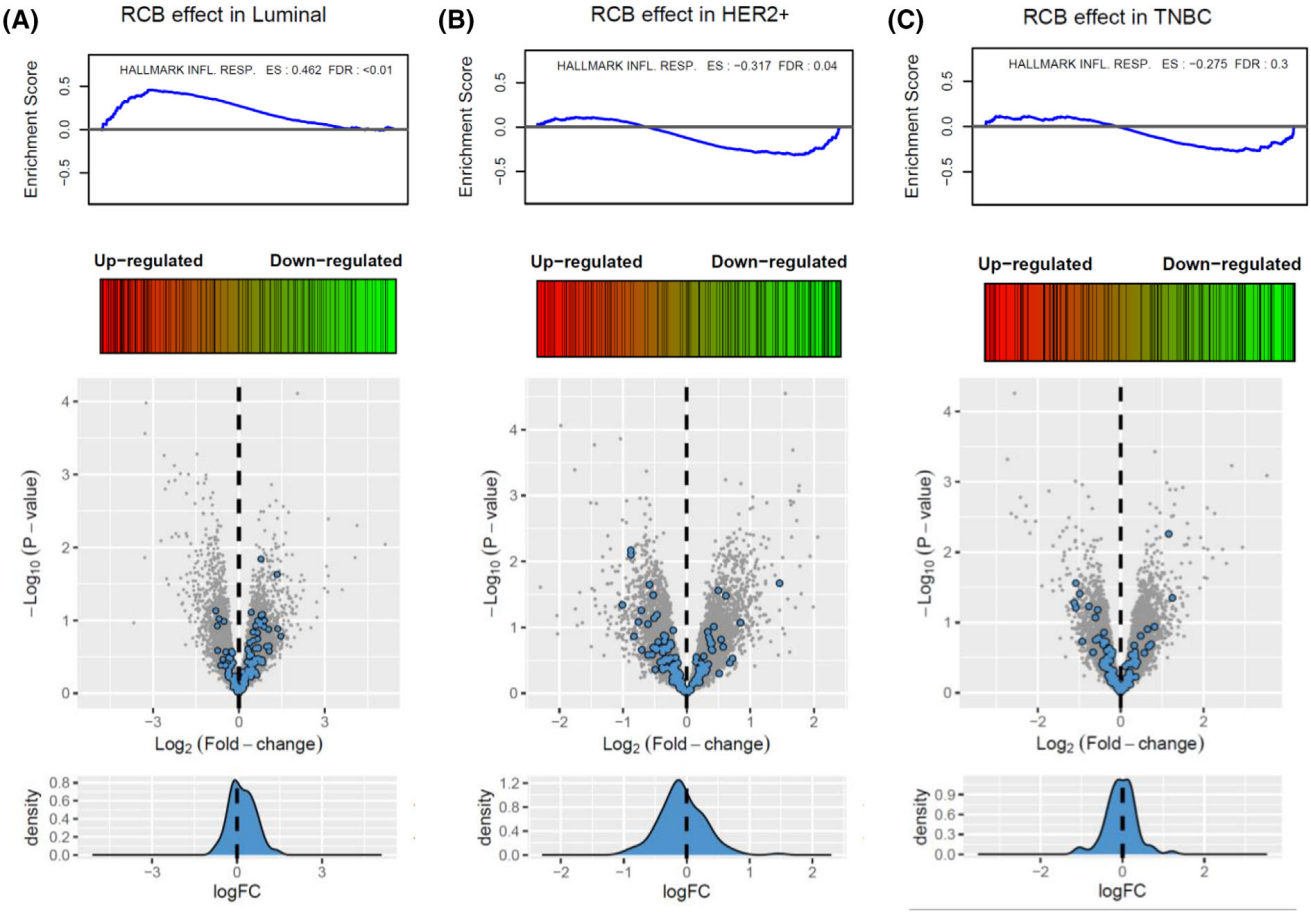
Enrichment of the inflammatory response hallmark gene set in diagnostic biopsies from BC patients not responding to NAC. Enrichment plots and volcano plots for the inflammatory response hallmark, when comparing non‐responding and responding tumors. (A) In luminal subtype; (B) In HER2+ subtype; (C) In triple negative subtype. BC, breast cancer; ES, enrichment score; FDR, false discovery rate; NAC, neoadjuvant chemotherapy; RCB, residual cancer burden.

In pre‐NAC samples, when comparing non‐responding to responding tumors in each subtype separately, inflammatory response‐related markers were significantly upregulated in luminal BC (ES >0.3, FDR <0.01) while being down‐regulated in HER2+ tumors (ES < −0.3, FDR <0.05). In the TNBC subtype, a non‐significant down‐regulation of inflammatory response‐related markers was seen (ES = −0.275, FDR = 0.3) (Fig. 4, Tables S12– S16). Further exploration of publicly available databases, GSE22226 and GSE25066, showed discordant results between subtypes (Fig. S5). In the GSE25066, analyzing 248 luminal breast cancers and 146 TNBC, we observed a significant upregulation of the inflammatory response‐related markers in the luminal and TNBC subtype. In the GSE22226, analyzing 23 luminal breast cancers, 20 HER2+, and 39 TNBC, we observed a significant downregulation of the inflammatory response‐related markers in the luminal subtype, whereas a significant upregulation was observed in the HER2+ subtype.

### 3.6 Higher percentage of TILs at diagnosis is seen in HER2+ BC and TNBC subtypes

**Fig. 5:**
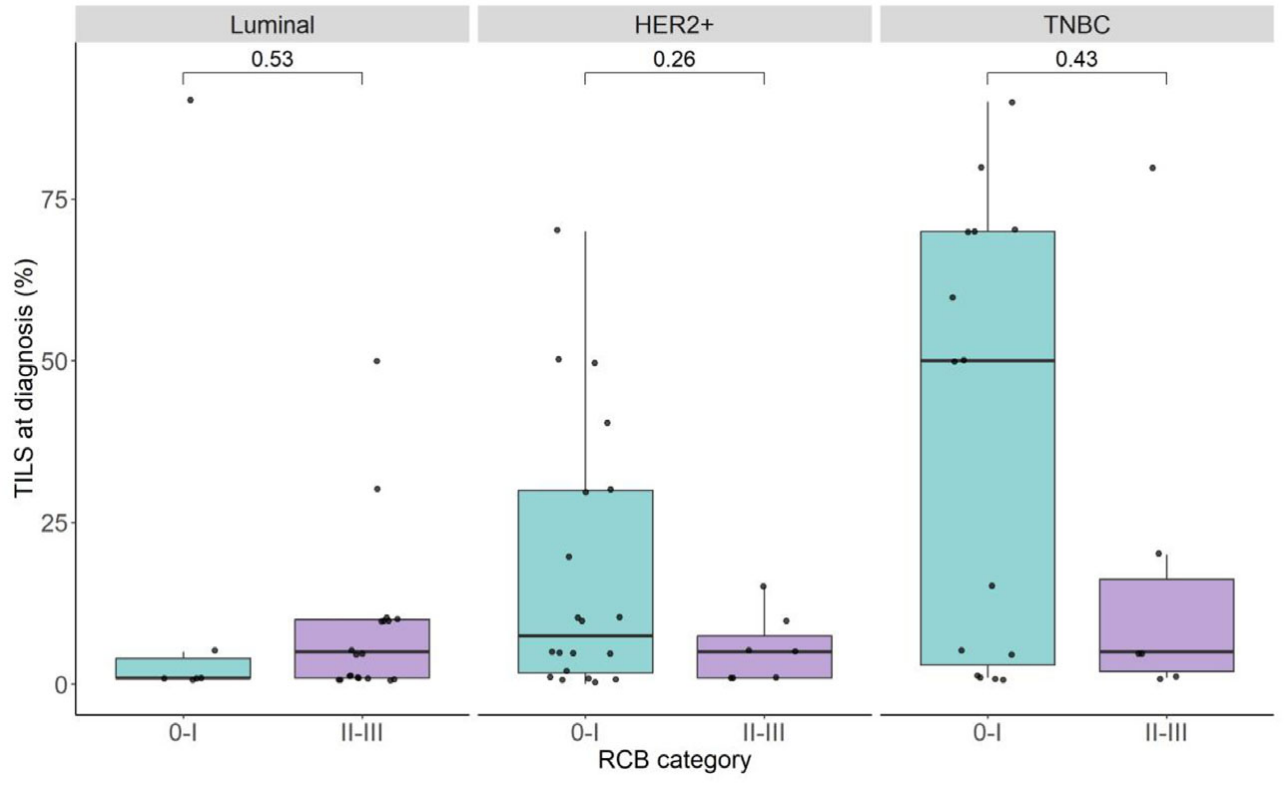
TILs evaluation at diagnosis according to the standardized method as described by the International Immuno‐oncology Biomarkers Working Group [11]. Comparison between responders and non‐responders in luminal subtype (RCB 0‐I: *n* = 6, RCB II‐III: *n* = 19); in HER2+ subtype (RCB 0‐I: *n* = 20, RCB II‐III: *n* = 7); in TNBC subtype (RCB 0‐I: *n* = 15, RCB II‐III: *n* = 7). Paired Wilcoxon test (±1.5 × IQR). RCB, residual cancer burden; TNBC, triple negative breast cancer; TILS, tumor‐infiltrating lymphocytes.

The extent of the stromal TILs was assessed in pre‐NAC samples (*n* = 73/100) according to the standardized method as described by the International Immuno‐oncology Biomarkers Working Group. We observed a trend towards a higher percentage of TILs in responders (RCB 0‐I) in comparison with non‐responders (RCB II‐III) in both HER2+ and TNBC, yet not statistically significant (Fig. 5).

### 3.7 In HER2+ BC subtype, responders have a higher percentage of CD3 + CD8‐FOXP3‐ lymphocytes at diagnosis

**Fig. 6:**
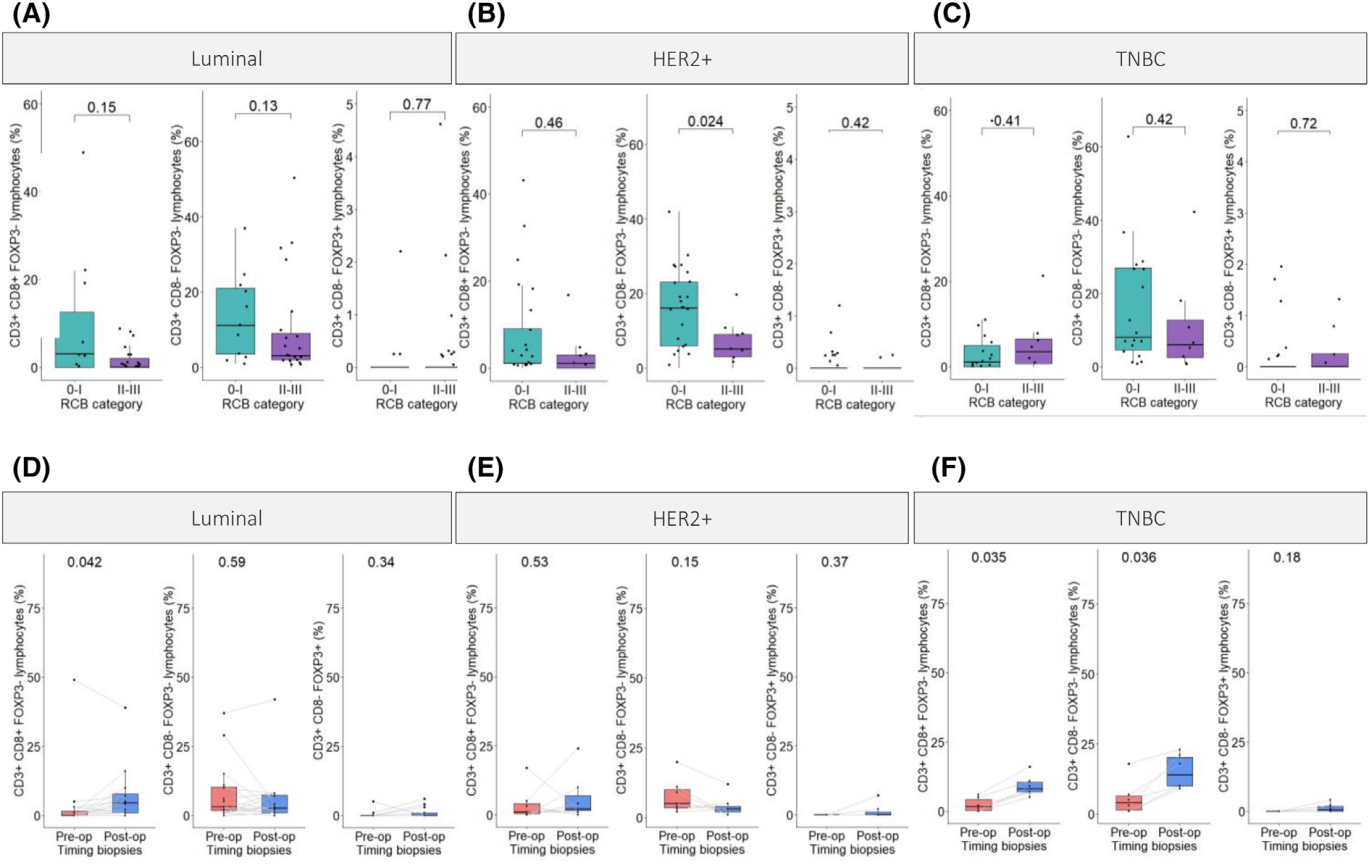
Abundance of lymphocyte populations within the stromal area of tumors from BC patients. Comparison between responders and non‐responders (A) in luminal subtype (RCB 0‐I: *n* = 11, RCB II‐III: *n* = 23); (B) in HER2+ subtype (RCB 0‐I: *n* = 25, RCB II‐III: *n* = 9); (C) in TNBC subtype (RCB 0‐I: *n* = 19, RCB II‐III: *n* = 8). For A, B, and C: Non‐paired Wilcoxon test (±1.5 × IQR). Paired comparison of pre‐NAC and post‐NAC samples (*n* = 29) (D) in luminal subtype in the stromal area (*n* = 16); (E) in HER2+ subtype in the stromal area (*n* = 7); (F) in TNBC subtype in the stromal area (*n* = 6). For D, E, and F: Paired Wilcoxon test (±1.5 × IQR). BC, breast cancer; NAC, neoadjuvant chemotherapy; RCB, residual cancer burden; TNBC, triple negative breast cancer.

We compared different types of TILs levels in both stroma and tumor clusters in pre‐NAC samples between responders and non‐responders (Fig. 6A–C; Fig. S6). No difference was observed regarding CD3+ CD8+ FOXP3‐ lymphocytes and CD3 + CD8‐FOXP3+ lymphocytes between responders and non‐responders in all subtypes. The levels of CD3+ CD8‐ FOXP3‐ lymphocytes were significantly higher in responders in the stroma of the HER2+ subgroup (*P*‐value 0.024) (Fig. 6B). When analyzing the spatial distribution of each subtype of lymphocytes at a distance of 25 μm from the tumor clusters, the same observation was made, while in at a distance of 50 μm we did not observe any difference between responders and non‐responders (Figs S7 and S8).

### 3.8 Lymphocyte infiltration increases in NAC‐treated residual tumors from luminal and TNBC subtypes

In paired analysis performed in cases with residual tumors, comparing pre‐NAC and post‐NAC samples, we observed a significant increase of CD3 + CD8 + FOXP3‐ lymphocytes in the stromal area of post‐NAC samples, in both luminal and TNBC subtypes (*P*‐values: 0.042 and 0.035, respectively) (Fig. 6D,F). The same observations were seen in the tumor cluster area (*P*‐values: 0.0041 and 0.035, respectively) (Fig. S9A,C). No significant difference in TILS levels was observed when comparing pre‐ and post‐NAC samples of the HER2+ BC subtype (Fig. 6E; Fig. S9A,C). In the TNBC subtype, a significant increase of CD3 + CD8‐FOXP3‐ lymphocytes was also noticed in both stromal and tumor areas from post‐NAC samples (*P*‐value 0.036 and 0.031, respectively) (Fig. 6F, Fig. S9A,C). No significant difference was observed in CD3 + CD8‐FOXP3+ lymphocytes between pre‐ and post‐NAC samples, even though this subpopulation showed a trend towards an increase in the stromal area in post‐NAC across all subtypes (Fig. 6D–F). When analyzing the spatial distribution of each subtype of lymphocytes at a distance of 25 and 50 μm from the tumor clusters, we observed a significantly higher percentage of CD3 + CD8 + FOXP3‐ lymphocytes in the microenvironment of the tumors in post‐operative samples for the luminal and TNBC subtypes and a significantly higher percentage of CD3 + CD8‐FOXP3‐ for the TNBC subtypes (Figs S10 and S11).

### 3.9 Correlation between TILs and EMT is observed in pre‐NAC samples at both RNA and protein levels

**Fig. 7:**
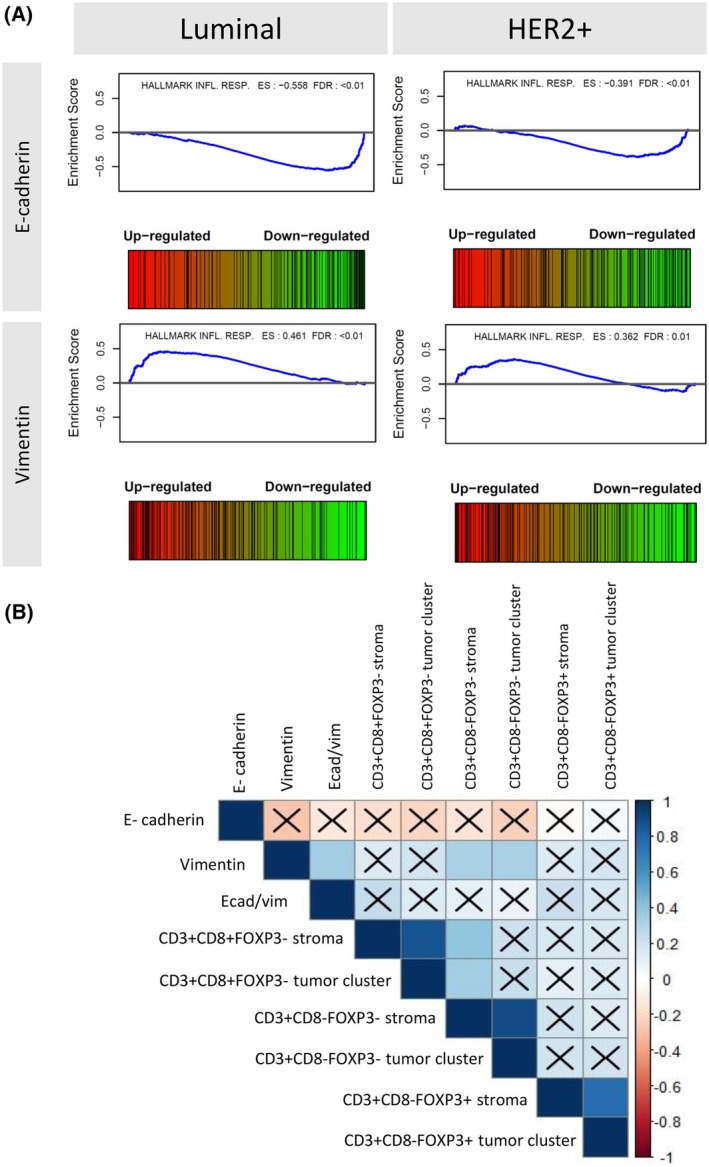
Correlation between EMT and TILS. (A). Enrichment plot of inflammatory response hallmark in pre‐NAC BC samples correlated with EMT marker levels. In both luminal and HER2+ BC subtypes, low levels of E‐cadherin and high levels of vimentin are correlated with an upregulation of the hallmark inflammatory response (ES, effect size; FDR, false discovery rate); (B) Correlation plot between EMT markers and TILs infiltration in diagnostic biopsies from BC patients. Low positive correlation is seen between vimentin levels and T‐helper lymphocyte abundance in both tumor and stromal areas. “X” represents a non‐significant *P*‐value. BC, breast cancer; ES, enrichment score; EMT, epithelial–mesenchymal transition; FDR, false discovery rate; NAC, neoadjuvant chemotherapy; TILS, tumor‐infiltrating lymphocytes.

Gene expression levels were then correlated with EMT markers obtained from multiplex IF in pre‐NAC samples across each molecular subtype, but the analysis could not reveal any significantly differentially expressed gene. However, GSEA revealed a downregulation of the inflammatory response gene hallmark in samples from luminal and HER2+ subtypes with a high E‐cadherin expression level (ES < −0.3, FDR <0.05), but also an upregulation of the same hallmark in the samples with a high vimentin expression level (ES >0.3, FDR <0.05) (Fig. 7A). Pearson's correlation test was performed to evaluate the correlation between EMT marker expression and the abundance of the different types of lymphocytes at the protein levels. We observed a significant low positive Pearson correlation coefficient between vimentin and CD3 + CD8‐FOXP3‐lymphocytes in the stromal area and the tumor cluster (*r* = 0.31 (*P*‐val = 0.002) and *r* = 0.32 (*P*val‐0.001), respectively) (Fig. 7B).

## 4 Discussion

In this trial, we analyzed EMT and lymphocyte infiltration in early BC specimens from diagnostic biopsies (pre‐NAC) of patients treated with NAC and surgical specimens (post‐NAC) from tumors with residual disease. The aim of this study was to investigate the predictive value of EMT and TILs in early BC tumors exposed to neoadjuvant chemotherapy (NAC) and to explore the evolution of EMT and TILs infiltration after NAC in residual tumors. We found that the EMT‐related gene hallmark was significantly upregulated in pre‐NAC samples of non‐responding tumors (RCB‐II and III) across all BC molecular subtypes, compared to responding tumors (RCB‐0 and I). To further explore EMT, multiplex IF was performed with two EMT markers, E‐cadherin and vimentin. The E‐cadherin protein plays a role in cell adhesion and acts as a tumor suppressor, and reduced E‐cadherin expression could be related to tumor invasion and metastatic escape [8]. Vimentin is a cytoskeleton protein of mesenchymal cells and is expressed in epithelial cells that undergo EMT. In pre‐NAC samples, we did not observe any difference in EMT at the protein level in non‐responders (RCB‐II and III) when compared to responders (RCB‐0 and I). Using the same markers, Elzamly et al. came to the same conclusion in a smaller cohort of early BC patients (all subtypes, *n* = 32) [23]. Masuda et al., who evaluated 64 paired TNBC samples using the same markers, showed that EMT could occur after NAC [24]. In another cohort of 80 paired samples with non‐luminal BC, a change in EMT markers was seen after NAC but in the opposite direction, with an increase of E‐cadherin expression and a decrease of vimentin at the mRNA and protein levels [25]. Interestingly, in our series of luminal BC (*n* = 16), a paired comparison of pre‐NAC and post‐NAC samples revealed a significant change of percentage of cells expressing E‐cadherin and vimentin towards EMT. To our knowledge, no paired comparison in this subtype has been performed in the past, EMT being essentially evaluated in non‐luminal breast cancer [26]. Moreover, we observed a significant increase of transition cells in residual tumors across all BC subtypes; this phenomenon was already described by Grasset et al., who showed that vimentin is essential for invasion but also that transition cells are required for colony formation [27]. At the RNA level, the inflammatory response hallmark was upregulated in RCB‐II and III tumors in the luminal BC subgroup, while being downregulated in the HER2+ subtype. We explored subpopulations of TILs using CD3, CD8 and FOXP3 in IF. In pre‐NAC samples of HER2+ cases, we detected a significantly higher percentage of CD3 + CD8‐FOXP3‐ lymphocytes in the stromal area of responding tumors, when compared to non‐responding patients. This trend was seen in all BC molecular subtypes separately. Comparing pre‐and post‐NAC samples of luminal BC, we observed a significant increase of CD3 + CD8 + FOXP3‐ lymphocytes in the post‐NAC samples, while in the TNBC subtype we observed significantly higher levels of both CD3 + CD8 + FOXP3‐ and CD3 + CD8‐FOXP3‐ lymphocytes in the post‐NAC samples. These results are consistent with previous reports showing that the use of cytotoxic chemotherapy could induce changes in the immune response against cancer cells [28]. Ruffel et al. already noticed an increase of the CD8/CD4 ratio after NAC in residual tumors, and Garcia‐Martinez et al. also reported a higher TILs infiltration in post‐NAC residual tumors [29, 30]. Several studies have shown a correlation between the increase of TILs in residual tumors after NAC and outcomes in both TNBC and HER2+ BC [31, 32, 33, 34, 35]. Finally, we observed an interplay between EMT and TILs infiltration at the RNA levels but also, although with a low correlation, at the protein level. This is in line with previous data showing that EMT and the tumor immune microenvironment are connected, as EMT could produce dynamic modifications of the immune cells by secreting different cytokines or chemokines, and as the immune infiltration is able to influence EMT towards the mesenchymal phenotype [36]. However, the correlation between this interplay and the response of early BC to NAC is still poorly understood.

This study has a few limitations: (a) the small sample size of our cohort, and therefore of each molecular subtype, limited multivariate analysis (i.e., logistic regression stratified in each molecular subtype) and the possibility to assess the added predictive value of RNA‐seq to classify non‐responding and responding tumors; (b) the sequencing of the post‐NAC samples was performed in a separate batch from the pre‐NAC samples, preventing the comparison of pre‐ and post‐NAC RNA‐seq data due to the confounding batch effect; (c) EMT is a complex cellular process characterized by several phases, and the choice of E‐cadherin and vimentin as EMT markers might not adequately capture the EMT process. Other EMT markers might be more accurate to study EMT at the protein level [37]. Long described as binary, this process is in fact a continuum of cell states characterized by several levels of epithelial and mesenchymal markers. Intermediate states–transitional states–exhibit distinct morphological, transcriptional, and epigenetic features in between epithelial and mesenchymal cells, and these intermediate states can be classified as early hybrid, hybrid, or late hybrid [38]. It has been shown that these transition states exhibit different markers by flow cytometry such as CD106, CD51, and CD61 but also different functional characteristics, with the early hybrid being more prompt to proliferate and form metastases and the late hybrid state having acquired increased capacity for invasion and plasticity. Even though recommendations suggest to evaluate EMT using different techniques and markers (flow cytometry, Western blot, immunohistochemistry, immunofluorescence), the research on EMT using human biopsies from patients with breast cancer is limited to the use of immunofluorescence with the use of E‐cadherin and vimentin in the majority of the studies [38]; (d) EMT is dynamic over time, and our results might not reflect the reality, since we only collected samples at diagnosis and after surgery. Nevertheless, our findings remain interesting because they highlight the relevance of EMT and TILs evaluation in early BC to better understand BC response or resistance to NAC. These results might therefore be useful for future studies exploring personalized NAC treatment and to further explore de‐ or escalation treatment strategies.

## 5 Conclusion

Altogether, our data show that EMT occurs and that subpopulations of TILs vary in non‐responding tumors. Being able to assess EMT and subpopulations of TILs at diagnosis or in residual tumors could lead to an escalation strategy in NAC or in adjuvant settings to improve response to therapy and later on, to improve the outcomes of patients. Before implementing these markers in daily diagnostic practice, our findings need to be validated in larger prospective cohorts.

## Conflict of interest

FD: consulting fee from Novartis. Support for meetings or travel from Novartis, Gilead. Advisory board from Novartis. Honoraria for presentations from Novartis and Pfizer. MVB: M.R. Van Bockstal previously received research support from Roche and Sysmex, as well as institutional consulting fees for being a member of advisory boards of AstraZeneca and Sakura, all unrelated to the present work. M.R. Van Bockstal received a postdoctoral clinical mandate (2019‐089) from the not‐for‐profit organization ‘Foundation Against Cancer’ (Brussels, Belgium) and is supported by the ‘Fonds Gaëtan Lagneaux’ (Fondation Saint‐Luc, Brussels, Belgium). GJ: consulting fees from Novartis, Amgen, Roche, Pfizer, Bristol‐Myers Squibb, Lilly, Astra‐Zeneca, Daiichi‐Sankyo, Seagen, Diaccurate, Payment or honoraria from Novartis, Amgen, Roche, Pfizer, Bristol‐Myers Squibb, Lilly, Daichii‐Sankyo, and Seagen. Support for meetings or travel from Novartis, Roche, Gilead, Pfizer, Lilly, Amgen, Bristol‐Myers Squibb, and Astra‐Zeneca. Advisory board from Novartis, Amgen, Roche, Pfizer, Bristol‐Myers Squibb, Lilly, Astra‐Zeneca, Daiichi‐Sankyo, and Seagen, Diaccurate. Receipt of equipment, materials, drugs, and medical writing from Novartis, Lilly, Roche, Amgen, Bristol‐Myers Squibb, and Astra‐Zeneca. FPD: Consulting or advisory role from Roche, Pfizer, AstraZeneca, Lilly, Novartis, Amgen, Daiichi Sankyo, Pierre Fabre, Gilead Sciences, Seagen, MSD oncology. Travel, accommodations, and expenses from Amgen, Roche, Teva, Pfizer, Daiichi Sankyo/AstraZeneca, and Gilead Sciences. JA, CVM, MB, CB, HD, ML, EB, VB, CJ, JT, AD, CB, CC: no conflict of interest.

## Author contributions

FPD, GJ, CJ, VB, CB conceived and designed the study. CB, AD, and HD developed the multiplex methodology and quantification. CJ, VB, JT, and GJ developed and performed the RNA extraction and sequencing methodology. FD, JA, CJ, EB, CVM, GJ, MB, CG, CC analyzed and interpreted the data. MVB and ML performed the TILs evaluation and quantification. FD, JA, CVM, MB, MVB, CG, GJ, VB, CJ, JT, AD, CB, CC, FPD wrote, reviewed, and revised the manuscript. All authors have read and approved the manuscript.

## Peer review

The peer review history for this article is available at https://www.webofscience.com/api/gateway/wos/peer‐review/10.1002/1878‐0261.13813.

## Supporting information


**Fig. S1.** Illustration of EMT and immune cells staining quantification on a whole tissue section of breast cancer stained by multiplex immunofluorescence (mIF) and immunohistochemistry (IHC). (A) FFPE sections were sequentially stained by mIF with an antibodies against vimentin, E‐cadherin, CD3, CD8 and Foxp3, followed by the Hoechst nuclear marker. (B) After whole slide fluorescence image acquisitions. (C) IHC was performed with a tumor marker using an antibody against pan‐cytokeratin CKAE1‐AE3 (CK, brown signal) on the same slide and (D) digitalized with the same slide scanner. (E) Tumor regions were manually circled by a Pathologist and (F) automatically adjusted to the tissue borders. (G) CK‐positive tumor regions were automatically delineated from CK‐negative stroma. (H) These tumor regions, detected on the brightfield scan, were transposed to the aligned fluorescent scan with the Visiopharm Tissue Align module. (I) Cells were detected in these regions using cell segmentation and classification of the Visiopharm software. EMT, epithelial‐mesenchymal transition; FFPE, Formalin‐fixed paraffin‐embedded.

**Fig. S2.** Enrichment of the EMT hallmark gene set in BC patients not responding to NAC in publicly available datasets GSE22226 and GSE25066. In the two datasets, comparison between non‐responders (RCB‐II and III) and reponders (RCB‐0 and I) have been done. For the GSE22226, in the luminal subtype 23 samples were analyzed (non‐responders: 19 and responders: 4), in the HER2+ subtype 20 samples were analyzed (non‐responders: 5 and responders: 15), in the TNBC subtype 39 samples were analyzed (non‐responders: 25 and responders: 14). For the GSE25066, in the luminal subtype 248 samples were analyzed (non‐responders: 202 and responders: 46), not enough sample were available in the HER2+ subtype and in the TNBC subtype 146 samples were analyzed (non‐responders: 86 and responders: 60). BC, breast cancer; EMT, epithelial‐mesenchymal transition; ES, enrichment score; FDR, false discovery rate; NAC, neoadjuvant chemotherapy; RCB, residual cancer burden; TNBC, triple negative breast cancer.

**Fig. S3.** Staining index evaluation for E‐cadherin and vimentin. (A, B) Comparison between responders and non‐responders for E‐cadherin and vimentin staining index – in luminal subtype (RCB 0‐I: *n* = 11, RCB II‐III: *n* = 22); in HER2+ subtype (RCB 0‐I: *n* = 24, RCB II‐III: *n* = 9); in TNBC subtype (RCB 0‐I: *n* = 17, RCB II‐III: *n* = 8). Non‐paired Wilcoxon test (±1.5 × IQR). (C, D) Paired comparison of pre‐NAC and post‐NAC samples for E‐cadherin and vimentin staining index — in luminal subtype (*n* = 16); in HER2+ subtype (*n* = 7); in TNBC subtype (*n* = 6). Paired Wilcoxon test (±1.5 × IQR). NAC, neoadjuvant chemotherapy; RCB, residual cancer burden; TNBC, triple negative breast cancer.

**Fig. S4.** Expression levels for EMT protein markers without lobular breast cancer. Comparison between responders and non‐responders (A) in luminal subtype (RCB 0‐I: *n* = 11, RCB II‐III: *n* = 19); (B) in HER2+ subtype (RCB 0‐I: *n* = 25, RCB II‐III: *n* = 9); (C) in TNBC subtype (RCB 0‐I: *n* = 18, RCB II‐III: *n* = 7). For A, B, C: Non‐paired Wilcoxon test (±1.5 × IQR). Paired comparison of E‐cadherin, vimentin and transition cells in pre‐NAC and post‐NAC samples (*n* = 24) (D) in luminal subtype (*n* = 12); (E) in HER2+ subtype (*n* = 7); (F) in TNBC subtype (*n* = 5). For D, E, F: Paired Wilcoxon test (±1.5 × IQR). EMT, epithelial‐mesenchymal transition; NAC, neoadjuvant chemotherapy; RCB, residual cancer burden; TNBC, triple negative breast cancer.

**Fig. S5.** Enrichment of the inflammatory response hallmark gene set in BC patients not responding to NAC in publicly available datasets GSE22226 and GSE25066. In the two datasets, comparison between non‐responders (RCB‐II and III) and reponders (RCB‐0 and I) have been done. For the GSE22226, in the luminal subtype 23 samples were analyzed (non‐responders: 19 and responders: 4), in the HER2+ subtype 20 samples were analyzed (non‐responders: 5 and responders: 15), in the TNBC subtype 39 samples were analyzed (non‐responders: 25 and responders: 14). For the GSE25066, in the luminal subtype 248 samples were analyzed (non‐responders: 202 and responders: 46), not enough sample were available in the HER2+ subtype and in the TNBC subtype 146 samples were analyzed (non‐responders: 86 and responders: 60). BC, breast cancer; ES, enrichment score; FDR, false discovery rate; NAC, neoadjuvant chemotherapy; RCB, residual cancer burden; TNBC, triple negative breast cancer.

**Fig. S6.** Percentage of lymphocytes in BC patients responding or not to NAC in the tumor cluster area. (A) In luminal subtype (RCB 0‐I: *n* = 11, RCB II‐III: *n* = 23); (B) HER2+ subtype (RCB 0‐I: *n* = 25, RCB II‐III: *n* = 9); (C) in TNBC subtype (RCB 0‐I: *n* = 19, RCB II‐III: *n* = 8). Non‐paired Wilcoxon test (±1.5×IQR). BC, breast cancer; NAC, neoadjuvant chemotherapy; RCB, residual cancer burden; TNBC, triple negative breast cancer.

**Fig. S7.** Percentage of lymphocytes in BC patients responding or not to NAC in the microenvironment at a distance of 25 μm from tumor clusters, for each molecular subtype. (A) Comparison in percentage between RCB 0‐I and RCB II‐III for the each subtypes of lymphocytes (luminal RCB 0‐I *n* = 11, RCB II‐III *n* = 23; HER2+ RCB 0‐I *n* = 25, RCB II‐III *n* = 9; TNBC RCB 0‐I *n* = 19, RCB II‐III *n* = 8). (B) Comparison of the distribution of each subtypes of lymphocytes per molecular subtypes of BC in the RCB 0‐I and RCB II‐III. Non‐paired Wilcoxon test (±1.5×IQR). BC, breast cancer; NAC, neoadjuvant chemotherapy; RCB, residual cancer burden; TNBC, triple negative breast cancer.

**Fig. S8.** Percentage of lymphocytes in BC patients responding or not to NAC in the microenvironment at a distance of 50 μm from tumor clusters, for each molecular subtype. (A) Comparison in percentage between RCB 0‐I and RCB II‐III for the each subtypes of lymphocytes (luminal RCB 0‐I *n* = 11, RCB II‐III *n* = 23; HER2+ RCB 0‐I *n* = 25, RCB II‐III *n* = 9; TNBC RCB 0‐I *n* = 19, RCB II‐III *n* = 8). (B) Comparison of the distribution of each subtypes of lymphocytes per molecular subtypes of BC in the RCB 0‐I and RCB II‐III. Non‐paired Wilcoxon test (±1.5×IQR). BC, breast cancer; NAC, neoadjuvant chemotherapy; RCB, residual cancer burden; TNBC, triple negative breast cancer.

**Fig. S9.** Paired comparison of pre‐ and post‐NAC samples in the tumor cluster area. (A) In luminal subtype in the stromal area (*n* = 16); (B) in HER2+ subtype in the stromal area (*n* = 7); (C) in TNBC subtype in the stromal area (*n* = 6). Paired Wilcoxon test (±1.5 × IQR). BC, breast cancer; NAC, neoadjuvant chemotherapy; RCB, residual cancer burden; TNBC, triple negative breast cancer.

**Fig. S10.** Paired comparison of pre‐ and post‐NAC samples of the percentage of lymphocytes in BC patients in the microenvironment at a distance of 25 μm from tumor clusters, in each molecular subtype. (A) CD3+CD8+FOXP3‐ in each molecular subtype; (B) CD3+CD8‐FOXP3‐ in each molecular subtype; (C) CD3+CD8‐FOXP3+ in each molecular subtype. Paired Wilcoxon test (±1.5 × IQR). BC, breast cancer; NAC, neoadjuvant chemotherapy.

**Fig. S11.** Paired comparison of pre‐ and post‐NAC samples of the percentage of lymphocytes in BC patients in the microenvironment at a distance of 50 μm from tumor clusters, in each molecular subtype. (A) CD3+CD8+FOXP3‐ in each molecular subtype; (B) CD3+CD8‐FOXP3‐ in each molecular subtype; (C) CD3+CD8‐FOXP3+ in each molecular subtype. Paired Wilcoxon test (±1.5 × IQR). BC, breast cancer; NAC, neoadjuvant chemotherapy.

**Table S1.** Antibodies used in the multiplex.

**Table S2.** Differentially expressed genes in luminal breast cancer when comparing non‐responding patients to responding patients.

**Table S3.** Differentially expressed genes in HER2+ breast cancer when comparing non‐responding patients to responding patients.

**Table S4.** Differentially expressed genes in TNBC when comparing non‐responding patients to responding patients.

**Table S5.** List of hallmarks in HER2+ breast cancer when comparing non‐responding patients to responding patients.

**Table S6.** List of hallmarks in TNBC, when comparing non‐responding patients to responding patients.

**Table S7.** List of hallmarks in luminal cancer when comparing non‐responding patients to responding patients.

**Table S8.** Differentially expressed genes related to EMT in luminal breast cancer when comparing non‐responding patients to responding patients.

**Table S9.** Differentially expressed genes related to EMT in HER2+ breast cancer when comparing non‐responding patients to responding patients.

**Table S10.** Differentially expressed genes related to EMT in TNBC when comparing non‐responding patients to responding patients.

**Table S11.** Immunofluorescence and immunohistochemistry quantification for E‐cadherin, vimentin, transition cells, CD3 + CD8 + FOXP3‐lymphocytes, CD3 + CD8‐FOXP3‐lymphocytes, CD3 + CD8‐FOXP3+ lymphocytes, E‐cadherin staining index, vimentin staining index, and TILs.

**Table S12.** Differentially expressed genes related to inflammatory response in luminal breast cancer when comparing non‐responding patients to responding patients.

**Table S13.** Differentially expressed genes related to inflammatory response in HER2+ breast cancer when comparing non‐responding patients to responding patients.

**Table S14.** Differentially expressed genes related to inflammatory response in TNBC when comparing non‐responding patients to responding patients.

**Table S15.** Pearson correlation between EMT expression and lymphocyte infiltration.

**Table S16.** *P*‐value of Pearson's correlation between EMT expression and lymphocyte infiltration.

## Data Availability

The datasets analyzed during the current study are available in the GEO repositories: GSE240671. The underlying code for this study is not publicly available but may be made available to qualified researchers on reasonable request from the corresponding author.
