# Supplementary figures and images for: Response to neoadjuvant chemotherapy in early breast cancers is associated with epithelial–mesenchymal transition and tumor‐infiltrating lymphocytes
Source: Mol Oncol. 2025 Feb 6;19(8):2330–47. doi: 10.1002/1878-0261.13813 (PMC12330941; doi:10.1002/1878-0261.13813)

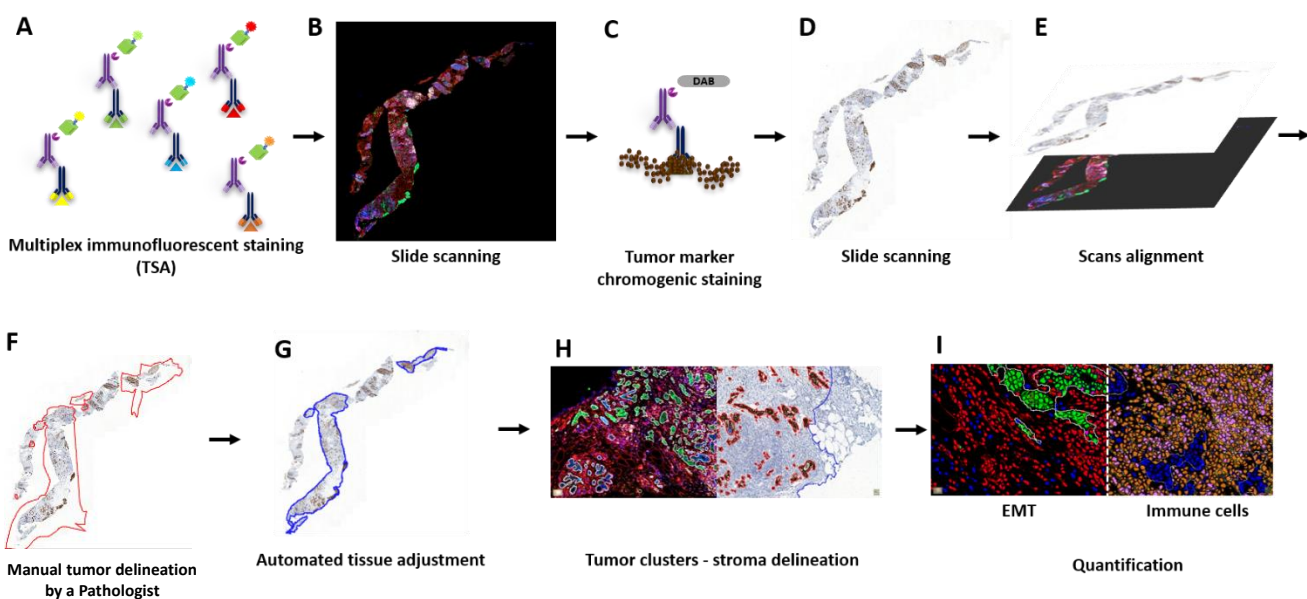

Supplement: Supplementary file 1 — Fig. S1. Illustration of EMT and immune cells staining quantification on a whole tissue section of breast cancer stained by multiplex immunofluorescence (mIF) and immunohistochemistry (IHC). (A) FFPE sections were sequentially stained by mIF with an antibodies against vimentin, E‐cadherin, CD3, CD8 and Foxp3, followed by the Hoechst nuclear marker. (B) After whole slide fluorescence image acquisitions. (C) IHC was performed with a tumor marker using an antibody against pan‐cytokeratin CKAE1‐AE3 (CK, brown signal) on the same slide and (D) digitalized with the same slide scanner. (E) Tumor regions were manually circled by a Pathologist and (F) automatically adjusted to the tissue borders. (G) CK‐positive tumor regions were automatically delineated from CK‐negative stroma. (H) These tumor regions, detected on the brightfield scan, were transposed to the aligned fluorescent scan with the Visiopharm Tissue Align module. (I) Cells were detected in these regions using cell segmentation and classification of the Visiopharm software. EMT, epithelial‐mesenchymal transition; FFPE, Formalin‐fixed paraffin‐embedded. [file MOL2-19-2330-s007.pdf]

Luminal

GSE22226

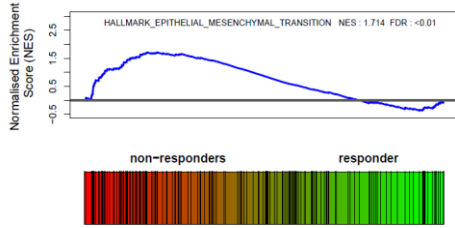

HER2+

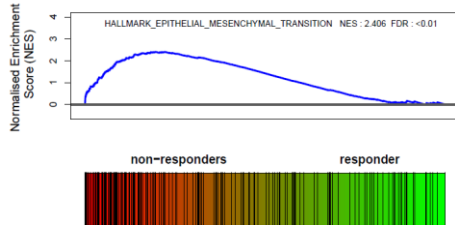

TNBC

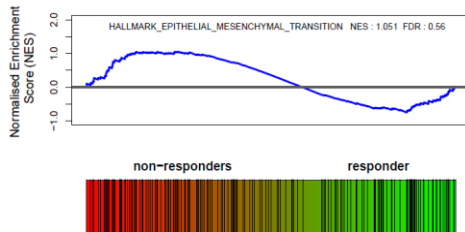

GSE25066

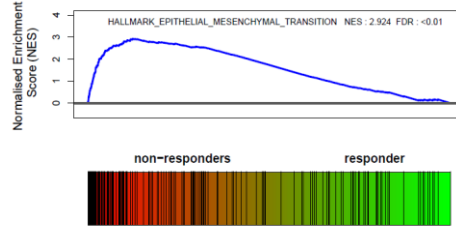

Not enough  
HER2+ samples  
available for  
the analysis

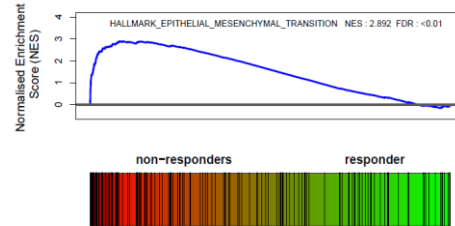

Supplement: Supplementary file 2 — Fig. S2. Enrichment of the EMT hallmark gene set in BC patients not responding to NAC in publicly available datasets GSE22226 and GSE25066. In the two datasets, comparison between non‐responders (RCB‐II and III) and reponders (RCB‐0 and I) have been done. For the GSE22226, in the luminal subtype 23 samples were analyzed (non‐responders: 19 and responders: 4), in the HER2+ subtype 20 samples were analyzed (non‐responders: 5 and responders: 15), in the TNBC subtype 39 samples were analyzed (non‐responders: 25 and responders: 14). For the GSE25066, in the luminal subtype 248 samples were analyzed (non‐responders: 202 and responders: 46), not enough sample were available in the HER2+ subtype and in the TNBC subtype 146 samples were analyzed (non‐responders: 86 and responders: 60). BC, breast cancer; EMT, epithelial‐mesenchymal transition; ES, enrichment score; FDR, false discovery rate; NAC, neoadjuvant chemotherapy; RCB, residual cancer burden; TNBC, triple negative breast cancer. [file MOL2-19-2330-s002.pdf]

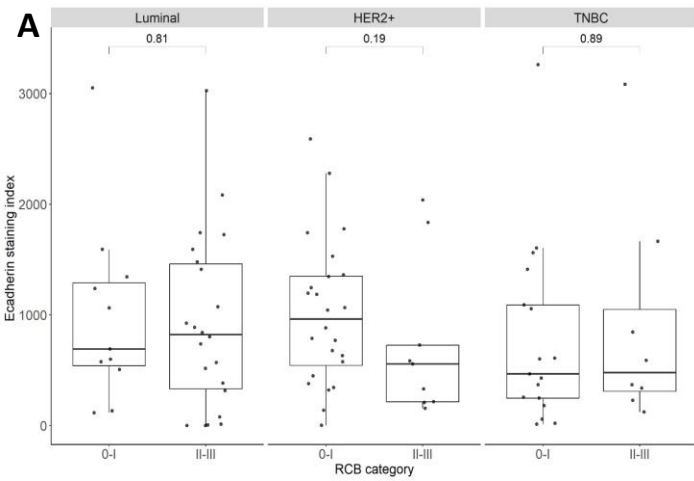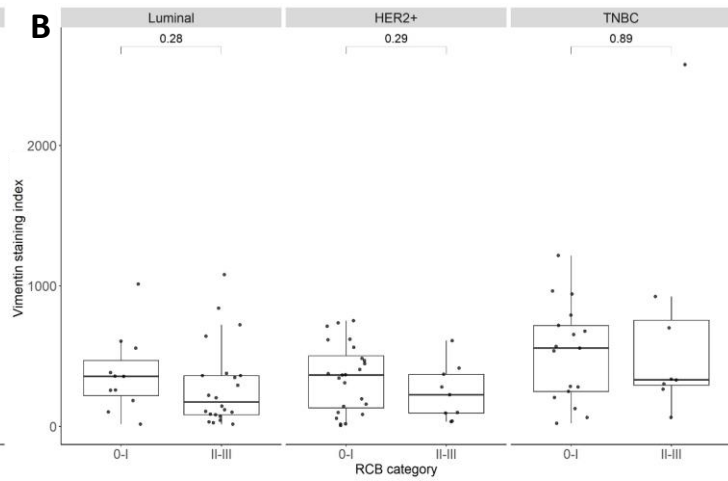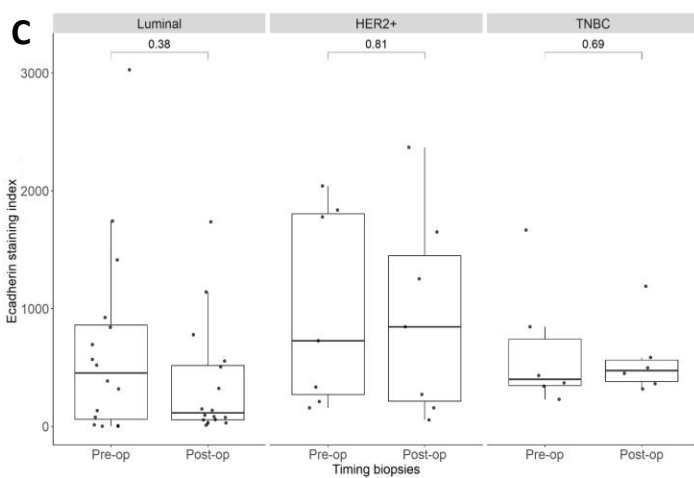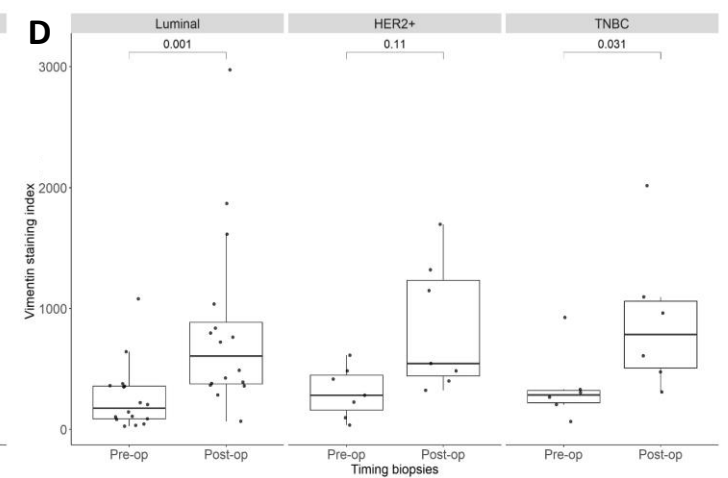

Supplement: Supplementary file 3 — Fig. S3. Staining index evaluation for E‐cadherin and vimentin. (A, B) Comparison between responders and non‐responders for E‐cadherin and vimentin staining index – in luminal subtype (RCB 0‐I: n = 11, RCB II‐III: n = 22); in HER2+ subtype (RCB 0‐I: n = 24, RCB II‐III: n = 9); in TNBC subtype (RCB 0‐I: n = 17, RCB II‐III: n = 8). Non‐paired Wilcoxon test (±1.5 × IQR). (C, D) Paired comparison of pre‐NAC and post‐NAC samples for E‐cadherin and vimentin staining index — in luminal subtype (n = 16); in HER2+ subtype (n = 7); in TNBC subtype (n = 6). Paired Wilcoxon test (±1.5 × IQR). NAC, neoadjuvant chemotherapy; RCB, residual cancer burden; TNBC, triple negative breast cancer. [file MOL2-19-2330-s015.pdf]

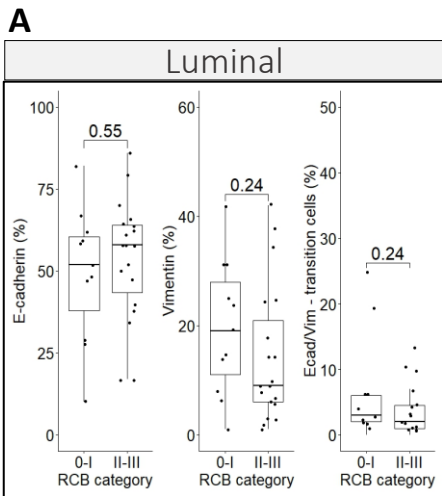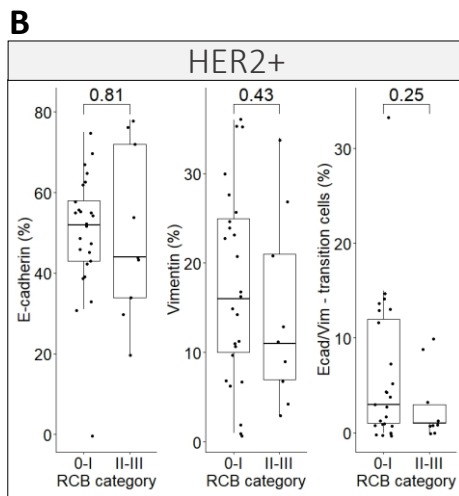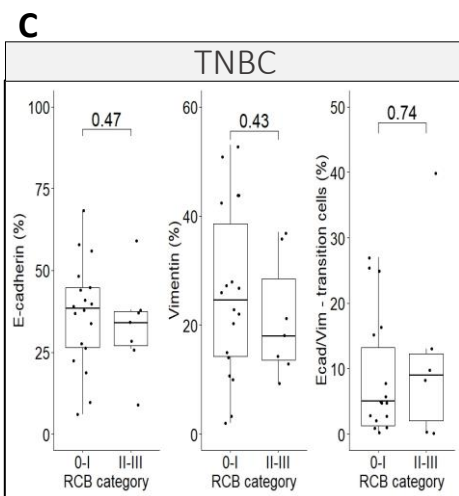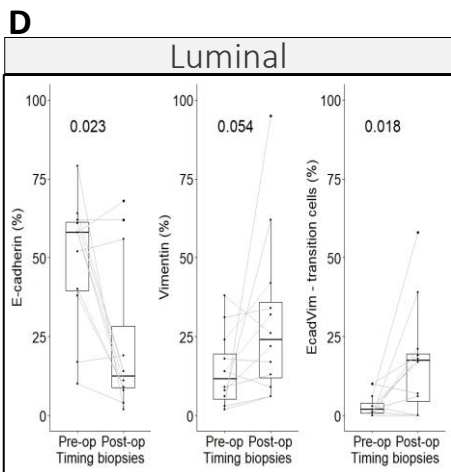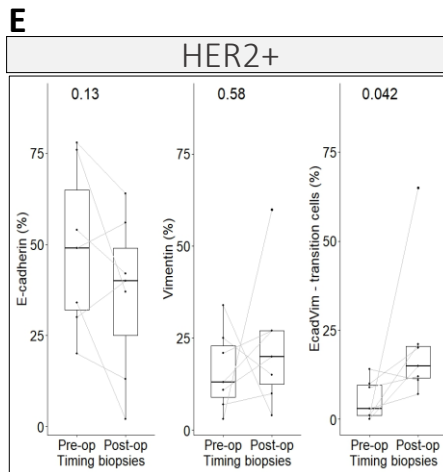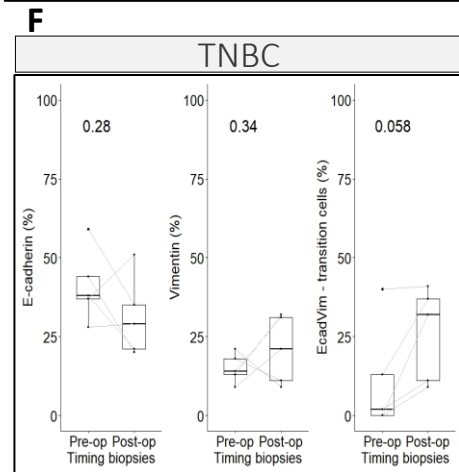

Supplement: Supplementary file 4 — Fig. S4. Expression levels for EMT protein markers without lobular breast cancer. Comparison between responders and non‐responders (A) in luminal subtype (RCB 0‐I: n = 11, RCB II‐III: n = 19); (B) in HER2+ subtype (RCB 0‐I: n = 25, RCB II‐III: n = 9); (C) in TNBC subtype (RCB 0‐I: n = 18, RCB II‐III: n = 7). For A, B, C: Non‐paired Wilcoxon test (±1.5 × IQR). Paired comparison of E‐cadherin, vimentin and transition cells in pre‐NAC and post‐NAC samples (n = 24) (D) in luminal subtype (n = 12); (E) in HER2+ subtype (n = 7); (F) in TNBC subtype (n = 5). For D, E, F: Paired Wilcoxon test (±1.5 × IQR). EMT, epithelial‐mesenchymal transition; NAC, neoadjuvant chemotherapy; RCB, residual cancer burden; TNBC, triple negative breast cancer. [file MOL2-19-2330-s019.pdf]

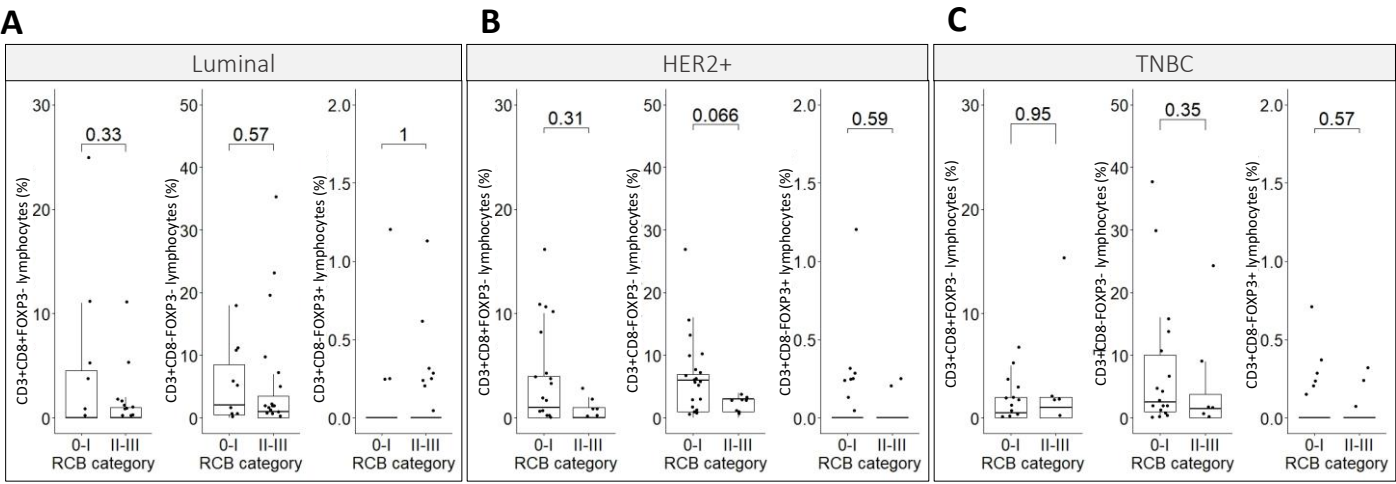

Supplement: Supplementary file 6 — Fig. S6. Percentage of lymphocytes in BC patients responding or not to NAC in the tumor cluster area. (A) In luminal subtype (RCB 0‐I: n = 11, RCB II‐III: n = 23); (B) HER2+ subtype (RCB 0‐I: n = 25, RCB II‐III: n = 9); (C) in TNBC subtype (RCB 0‐I: n = 19, RCB II‐III: n = 8). Non‐paired Wilcoxon test (±1.5×IQR). BC, breast cancer; NAC, neoadjuvant chemotherapy; RCB, residual cancer burden; TNBC, triple negative breast cancer. [file MOL2-19-2330-s013.pdf]

**A**

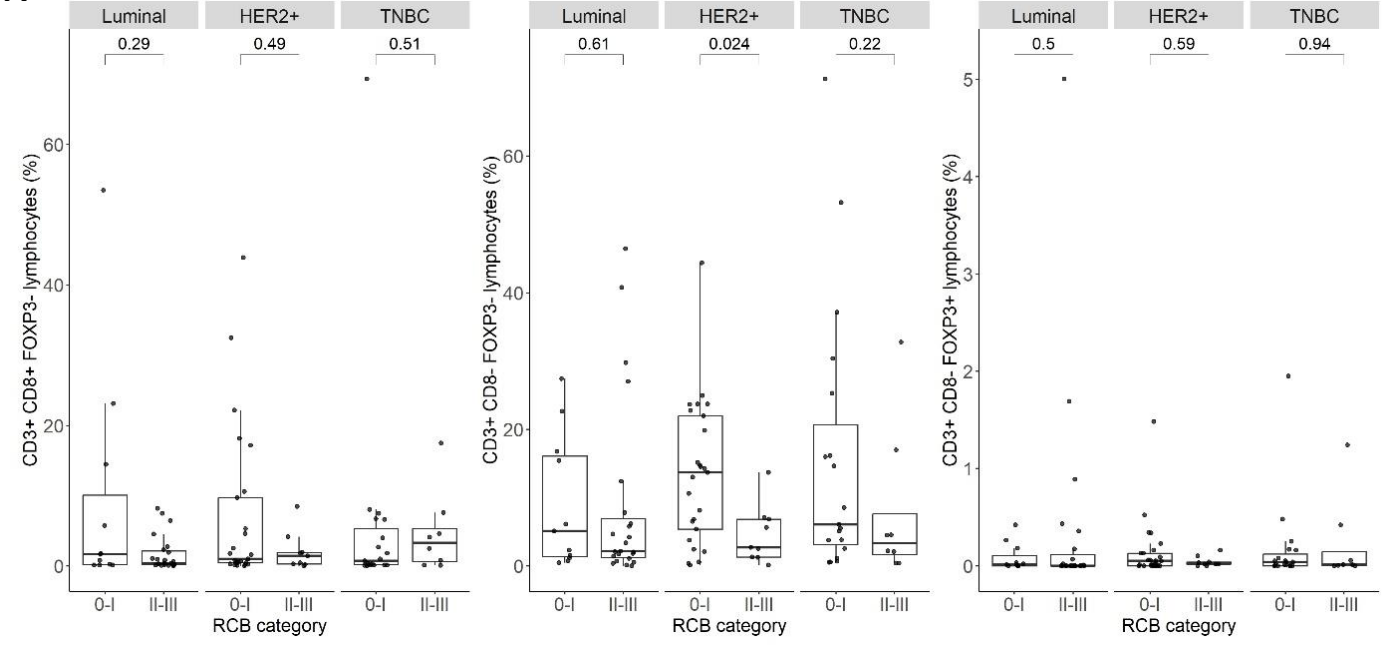

**B**

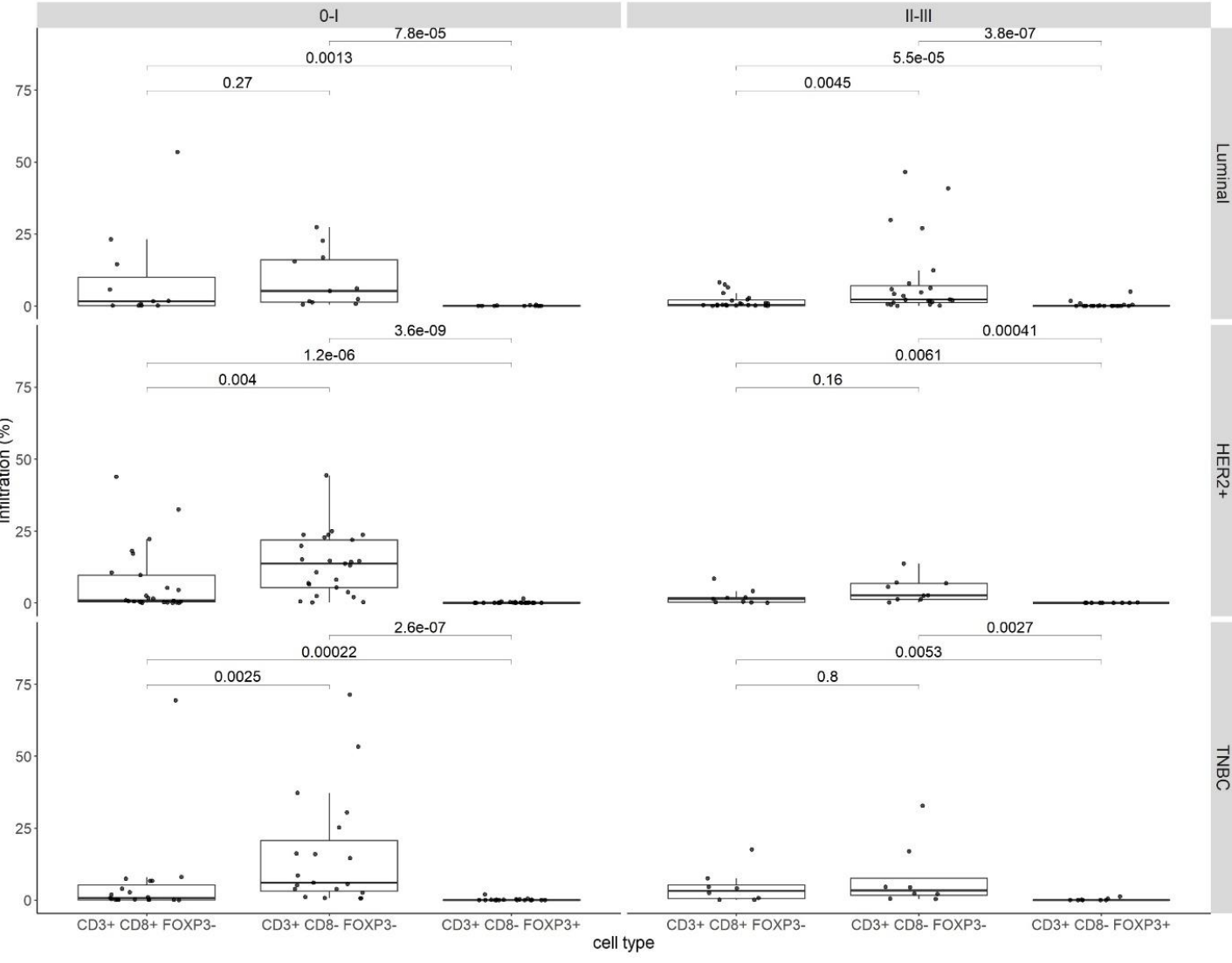

Supplement: Supplementary file 7 — Fig. S7. Percentage of lymphocytes in BC patients responding or not to NAC in the microenvironment at a distance of 25 μm from tumor clusters, for each molecular subtype. (A) Comparison in percentage between RCB 0‐I and RCB II‐III for the each subtypes of lymphocytes (luminal RCB 0‐I n = 11, RCB II‐III n = 23; HER2+ RCB 0‐I n = 25, RCB II‐III n = 9; TNBC RCB 0‐I n = 19, RCB II‐III n = 8). (B) Comparison of the distribution of each subtypes of lymphocytes per molecular subtypes of BC in the RCB 0‐I and RCB II‐III. Non‐paired Wilcoxon test (±1.5×IQR). BC, breast cancer; NAC, neoadjuvant chemotherapy; RCB, residual cancer burden; TNBC, triple negative breast cancer. [file MOL2-19-2330-s026.pdf]

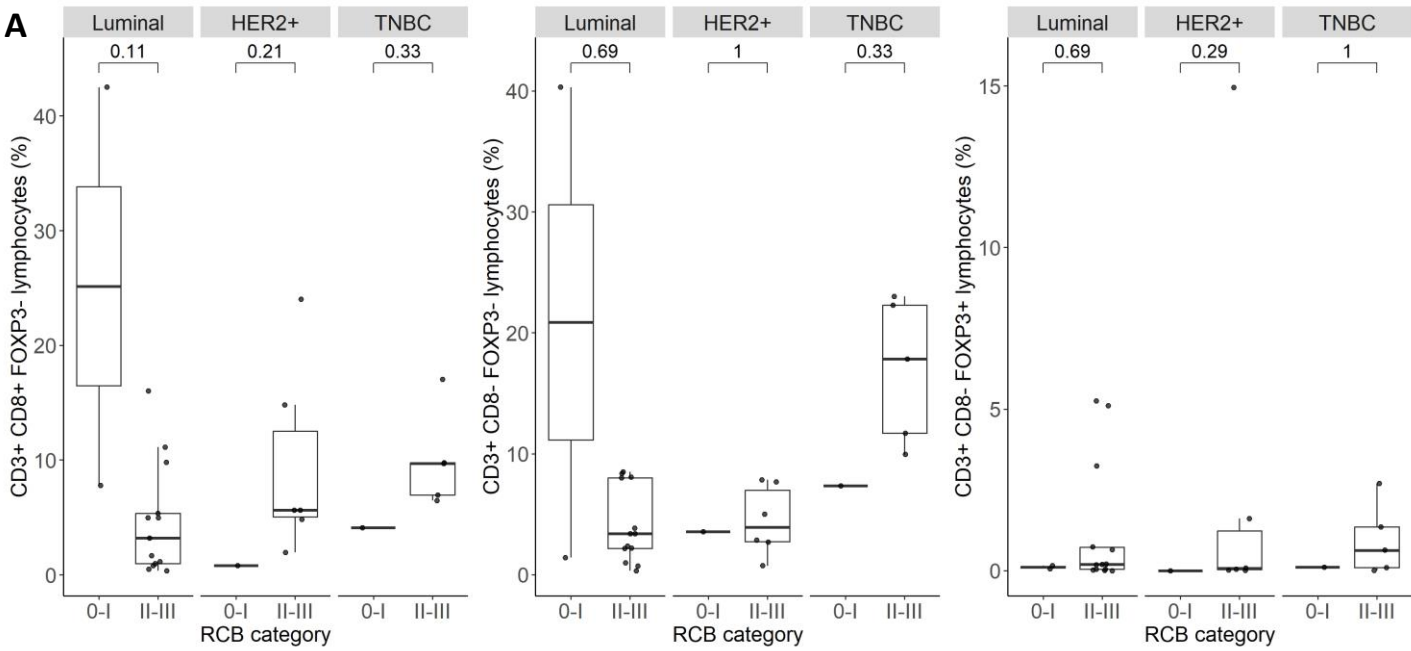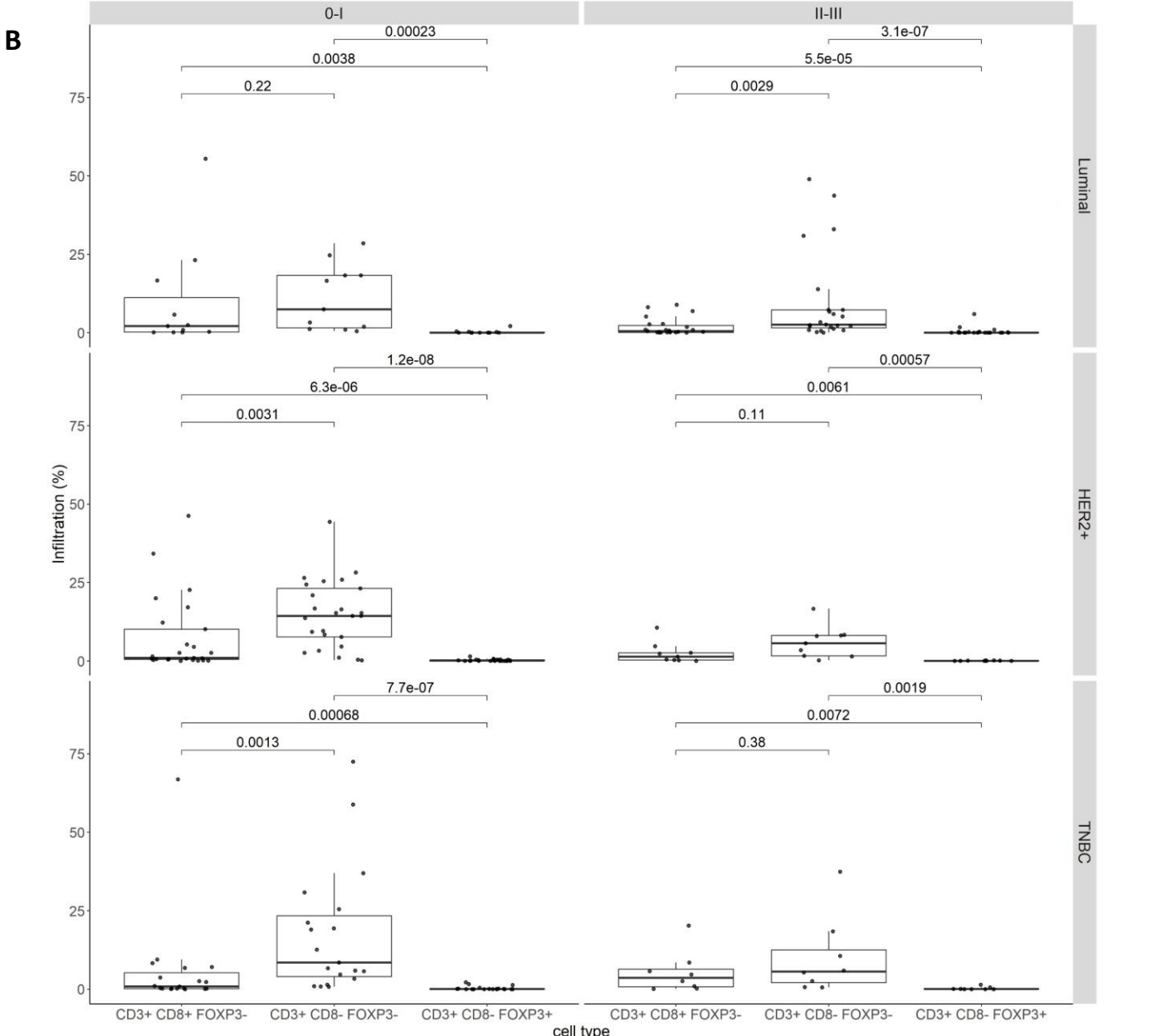

Supplement: Supplementary file 8 — Fig. S8. Percentage of lymphocytes in BC patients responding or not to NAC in the microenvironment at a distance of 50 μm from tumor clusters, for each molecular subtype. (A) Comparison in percentage between RCB 0‐I and RCB II‐III for the each subtypes of lymphocytes (luminal RCB 0‐I n = 11, RCB II‐III n = 23; HER2+ RCB 0‐I n = 25, RCB II‐III n = 9; TNBC RCB 0‐I n = 19, RCB II‐III n = 8). (B) Comparison of the distribution of each subtypes of lymphocytes per molecular subtypes of BC in the RCB 0‐I and RCB II‐III. Non‐paired Wilcoxon test (±1.5×IQR). BC, breast cancer; NAC, neoadjuvant chemotherapy; RCB, residual cancer burden; TNBC, triple negative breast cancer. [file MOL2-19-2330-s021.pdf]

**A**

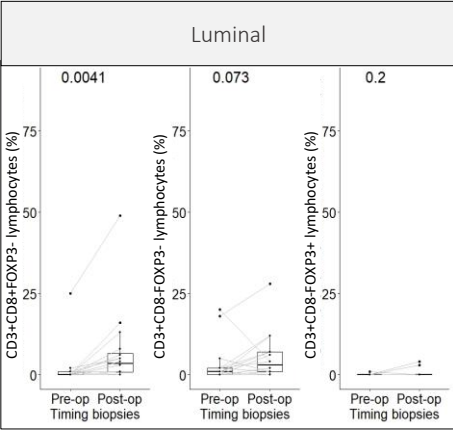

**B**

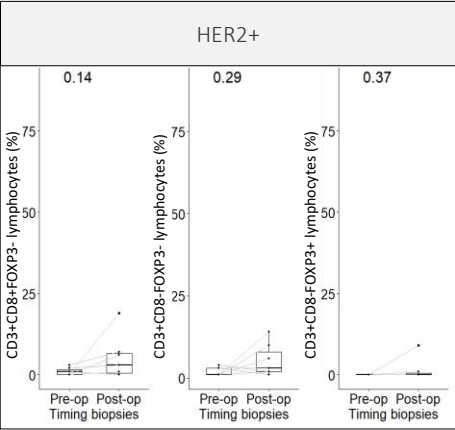

**C**

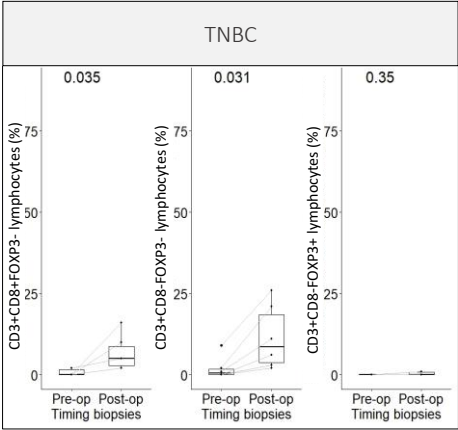

Supplement: Supplementary file 9 — Fig. S9. Paired comparison of pre‐ and post‐NAC samples in the tumor cluster area. (A) In luminal subtype in the stromal area (n = 16); (B) in HER2+ subtype in the stromal area (n = 7); (C) in TNBC subtype in the stromal area (n = 6). Paired Wilcoxon test (±1.5 × IQR). BC, breast cancer; NAC, neoadjuvant chemotherapy; RCB, residual cancer burden; TNBC, triple negative breast cancer. [file MOL2-19-2330-s006.pdf]

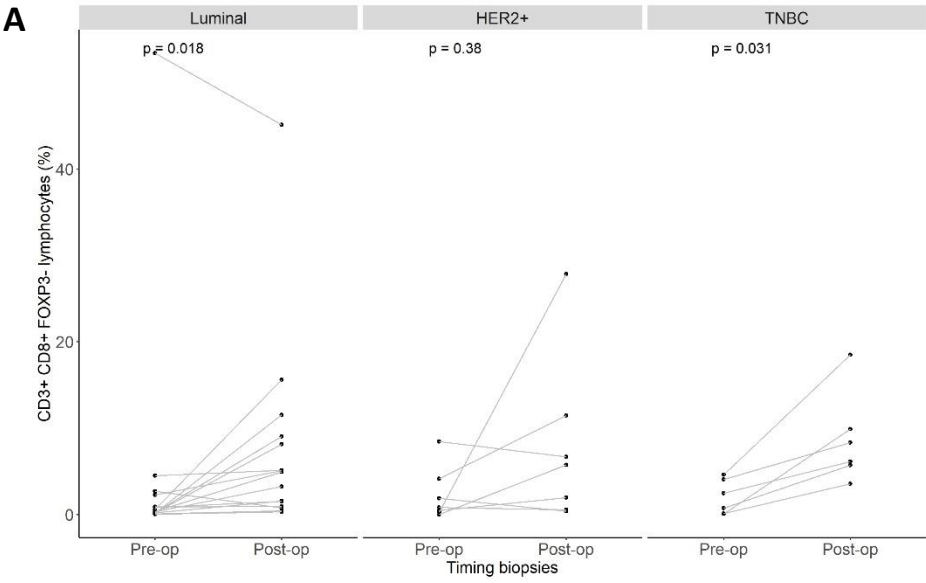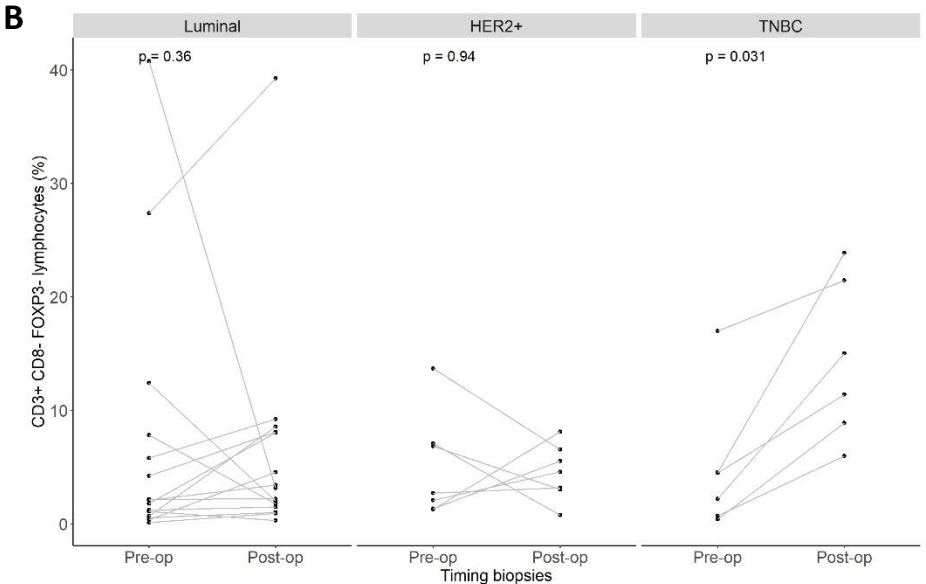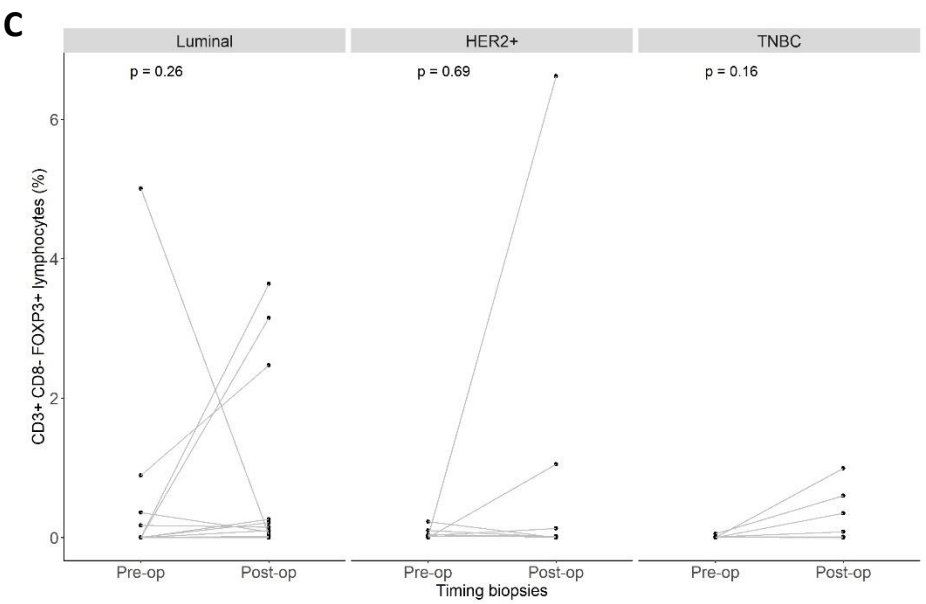

Supplement: Supplementary file 10 — Fig. S10. Paired comparison of pre‐ and post‐NAC samples of the percentage of lymphocytes in BC patients in the microenvironment at a distance of 25 μm from tumor clusters, in each molecular subtype. (A) CD3+CD8+FOXP3‐ in each molecular subtype; (B) CD3+CD8‐FOXP3‐ in each molecular subtype; (C) CD3+CD8‐FOXP3+ in each molecular subtype. Paired Wilcoxon test (±1.5 × IQR). BC, breast cancer; NAC, neoadjuvant chemotherapy. [file MOL2-19-2330-s025.pdf]

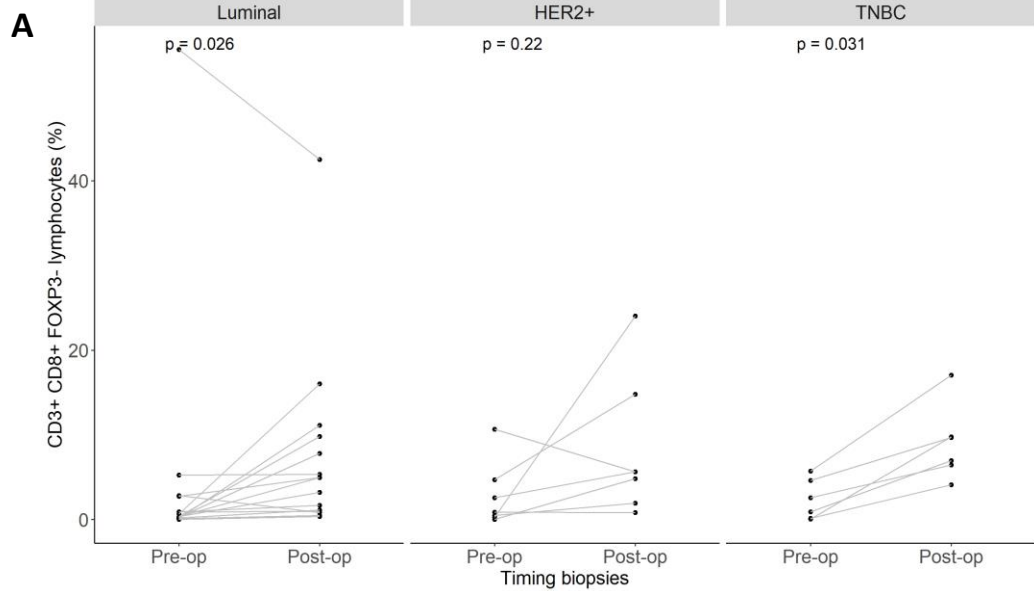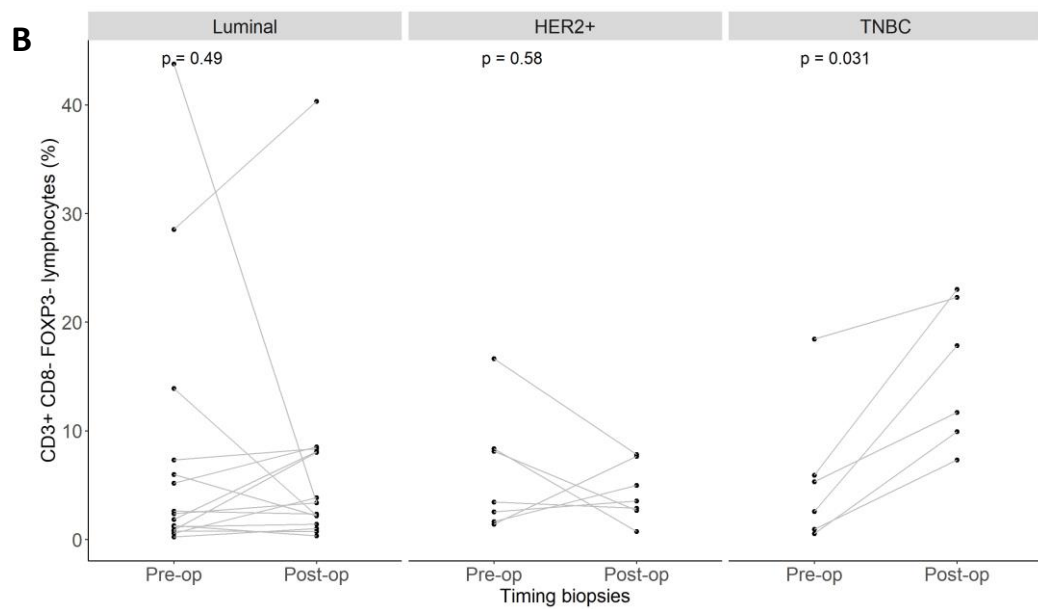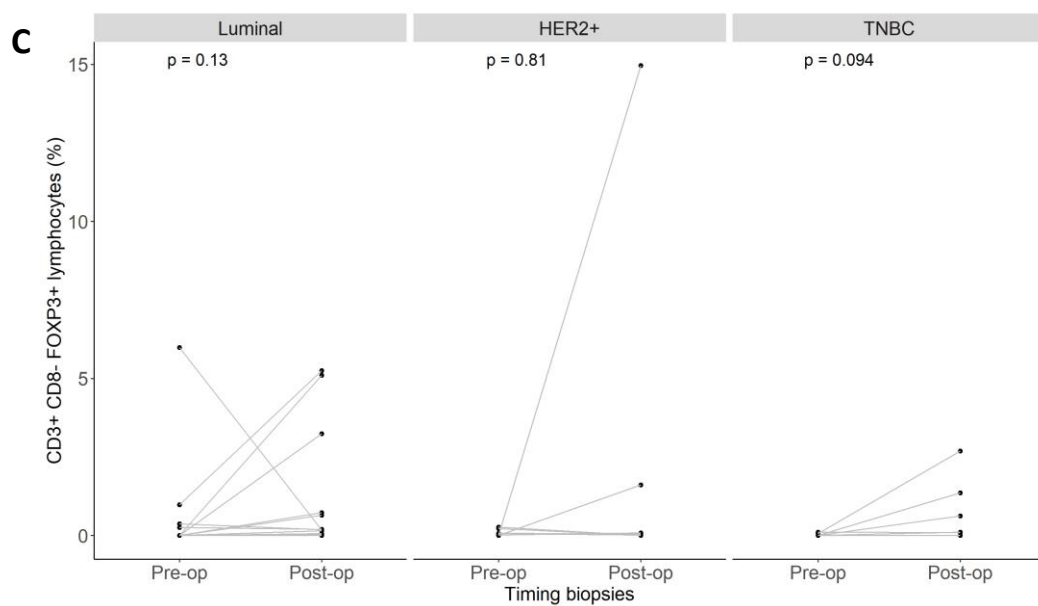

Supplement: Supplementary file 11 — Fig. S11. Paired comparison of pre‐ and post‐NAC samples of the percentage of lymphocytes in BC patients in the microenvironment at a distance of 50 μm from tumor clusters, in each molecular subtype. (A) CD3+CD8+FOXP3‐ in each molecular subtype; (B) CD3+CD8‐FOXP3‐ in each molecular subtype; (C) CD3+CD8‐FOXP3+ in each molecular subtype. Paired Wilcoxon test (±1.5 × IQR). BC, breast cancer; NAC, neoadjuvant chemotherapy. [file MOL2-19-2330-s008.pdf]
