# Supplementary material for: Response to neoadjuvant chemotherapy in early breast cancers is associated with epithelial–mesenchymal transition and tumor‐infiltrating lymphocytes
Source: Mol Oncol. 2025 Feb 6;19(8):2330–47. doi: 10.1002/1878-0261.13813 (PMC12330941; doi:10.1002/1878-0261.13813)
Supplement: Supplementary file 5 — Fig. S5. Enrichment of the inflammatory response hallmark gene set in BC patients not responding to NAC in publicly available datasets GSE22226 and GSE25066. In the two datasets, comparison between non‐responders (RCB‐II and III) and reponders (RCB‐0 and I) have been done. For the GSE22226, in the luminal subtype 23 samples were analyzed (non‐responders: 19 and responders: 4), in the HER2+ subtype 20 samples were analyzed (non‐responders: 5 and responders: 15), in the TNBC subtype 39 samples were analyzed (non‐responders: 25 and responders: 14). For the GSE25066, in the luminal subtype 248 samples were analyzed (non‐responders: 202 and responders: 46), not enough sample were available in the HER2+ subtype and in the TNBC subtype 146 samples were analyzed (non‐responders: 86 and responders: 60). BC, breast cancer; ES, enrichment score; FDR, false discovery rate; NAC, neoadjuvant chemotherapy; RCB, residual cancer burden; TNBC, triple negative breast cancer. [file MOL2-19-2330-s022.pdf]

	GSE22226	GSE25066
Luminal	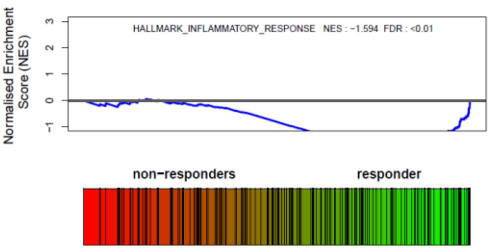	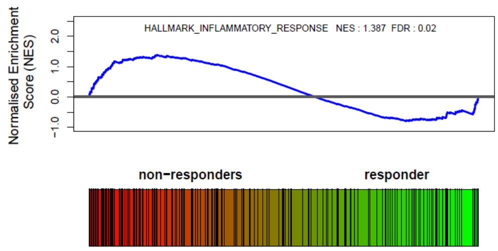
HER2+	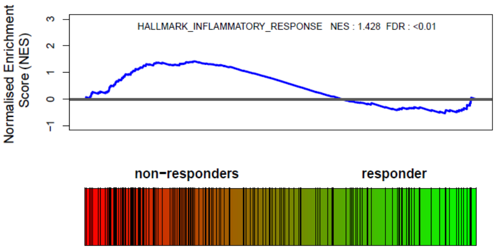	Not enough HER2+ samples available for the analysis
TNBC	No change in the hallmark inflammatory response	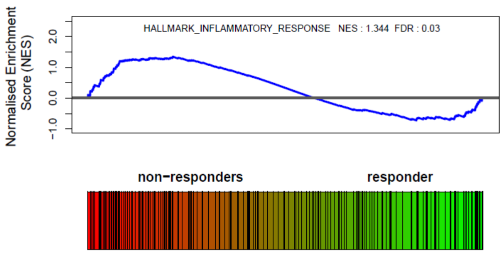
